# An extended and improved CCFv3 annotation and Nissl atlas of the entire mouse brain

**DOI:** 10.1162/imag_a_00565

**Published:** 2025-05-21

**Authors:** Sébastien Piluso, Csaba Verasztó, Harry Carey, Émilie Delattre, Thibaud L’Yvonnet, Éloïse Colnot, Armando Romani, Jan G. Bjaalie, Henry Markram, Daniel Keller

**Affiliations:** Blue Brain Project, École Polytechnique Fédérale de Lausanne (EPFL), Geneva, Switzerland; Department of Molecular Medicine, Institute of Basic Medical Sciences, University of Oslo, Oslo, Norway

**Keywords:** atlas, brain, registration, mouse, central nervous system

## Abstract

Brain atlases are indispensable tools for quantifying cellular composition across mouse brain regions. The widely used Common Coordinate Framework version 3 (CCFv3) from the Allen Institute delineates over 600 anatomical regions but lacks coverage of the most rostral and caudal brain areas, including the main olfactory bulb, cerebellum, and medulla. Additionally, the CCFv3 does not include annotations for key cerebellar layers, and its Nissl-stained reference volume is misaligned, limiting its efficiency. To overcome these limitations, we developed the Blue Brain Project (BBP) CCFv3 augmented atlas (CCFv3_BBP_), which includes a fully annotated mouse brain and an improved Nissl-stained reference volume aligned with the CCFv3_BBP_. This enhanced atlas also features the central nervous system annotation. Building on this enhanced resource, we aligned 734 Nissl-stained brains to generate an average Nissl template at 10 µm resolution. This new atlas version enabled the construction of the first comprehensive*in silico*model of cell distribution across the whole mouse central nervous system. This open-access resource broadens the applicability of brain atlases, supporting advancements in alignment accuracy, cell type mapping, and multimodal data integration.

## 1 Introduction

Reference atlases are essential tools for advancing the understanding of brain structure and function in both healthy and pathological contexts (Bjerke et al., 2018;Hawrylycz et al., 2014;Ng et al., 2007;Perens et al., 2023;Tward et al., 2020). Over the past several decades, numerous digital atlases of the rodent brain have been developed. Among these, the Allen Institute for Brain Science (AIBS) produced a widely used open-access atlas for the mouse brain in 2007 (Lein et al., 2007;Lau et al., 2008). Similarly, initiatives by the International Neuroinformatics Coordinating Facility, including the Waxholm Space atlas (G. A. Johnson et al., 2010;Papp et al., 2014), have contributed significantly to the field. These atlases have undergone continuous refinement, driven by advancements in optical microscopy and brain image analysis (Amato et al., 2016;H. J. Johnson et al., 2015;Milligan et al., 2019;Perens et al., 2023;Ragan et al., 2012;Voigt et al., 2019).

Most of these atlases rely on data from a limited number of brains (Bertrand & Nissanov, 2008;G. A. Johnson et al., 2010;Kovačević et al., 2005;Ma et al., 2008). In contrast, the AIBS’s Common Coordinate Framework (CCF) atlas stands out due to its derivation from a significantly larger dataset. Its latest version (CCFv3) is based on a serial two-photon tomography (STPT) average template constructed from 1,675 mouse brains (Kuan et al., 2015;Lein et al., 2007;Q. Wang et al., 2020). Over the past decade, the CCFv3 has become a standard for the mouse neuroscience community, facilitating classification and quantification of cells in the brain within a unified reference space (Chen et al., 2019;Chon et al., 2019;Ecker et al., 2017;Erö et al., 2018;Iqbal et al., 2019;Y. Kim et al., 2017;Leergaard & Bjaalie, 2022;Murakami et al., 2018;Rodarie et al., 2022;Shi et al., 2023;Yao et al., 2023;Zhang et al., 2021,^2023^). Its applications span a wide array of fields, including 2D histological analysis (Blixhavn et al., 2024;Carey et al., 2023;Piluso et al., 2024;Puchades et al., 2019;Sadeghi et al., 2023;Song et al., 2020;Tappan et al., 2019;Yates et al., 2019;Young et al., 2021), 3D histology leveraging light-sheet fluorescence microscopy (Liebmann et al., 2016;Perens et al., 2021,^2023^;Renier et al., 2016), and in other fields (Ortiz et al., 2020;N. Wang et al., 2023).

The CCF is an invaluable resource for the scientific community, especially for studying and modeling neuronal circuits in the brain. These qualities align with the initiative of the*Blue Brain Project*(BBP), which endeavors to reconstruct and simulate the entire mouse brain (Markram, 2006;Markram et al., 2015). Despite its wide utility, the CCFv3 has notable limitations. It does not encompass the entire brain, excluding key regions such as the olfactory bulb, cerebellum, and medulla. Furthermore, the atlas model lacks a representation of the spinal cord, preventing its application to the mouse central nervous system. Additionally, the alignment between the Allen Reference Atlas (ARA) Nissl version and the CCFv3 template remains imperfect (Rodarie et al., 2022). These misalignments limit the precision of single-modality registrations, particularly in the hippocampus, cerebellum, and other regions critical for neuroanatomical studies.

**Fig. 1.**
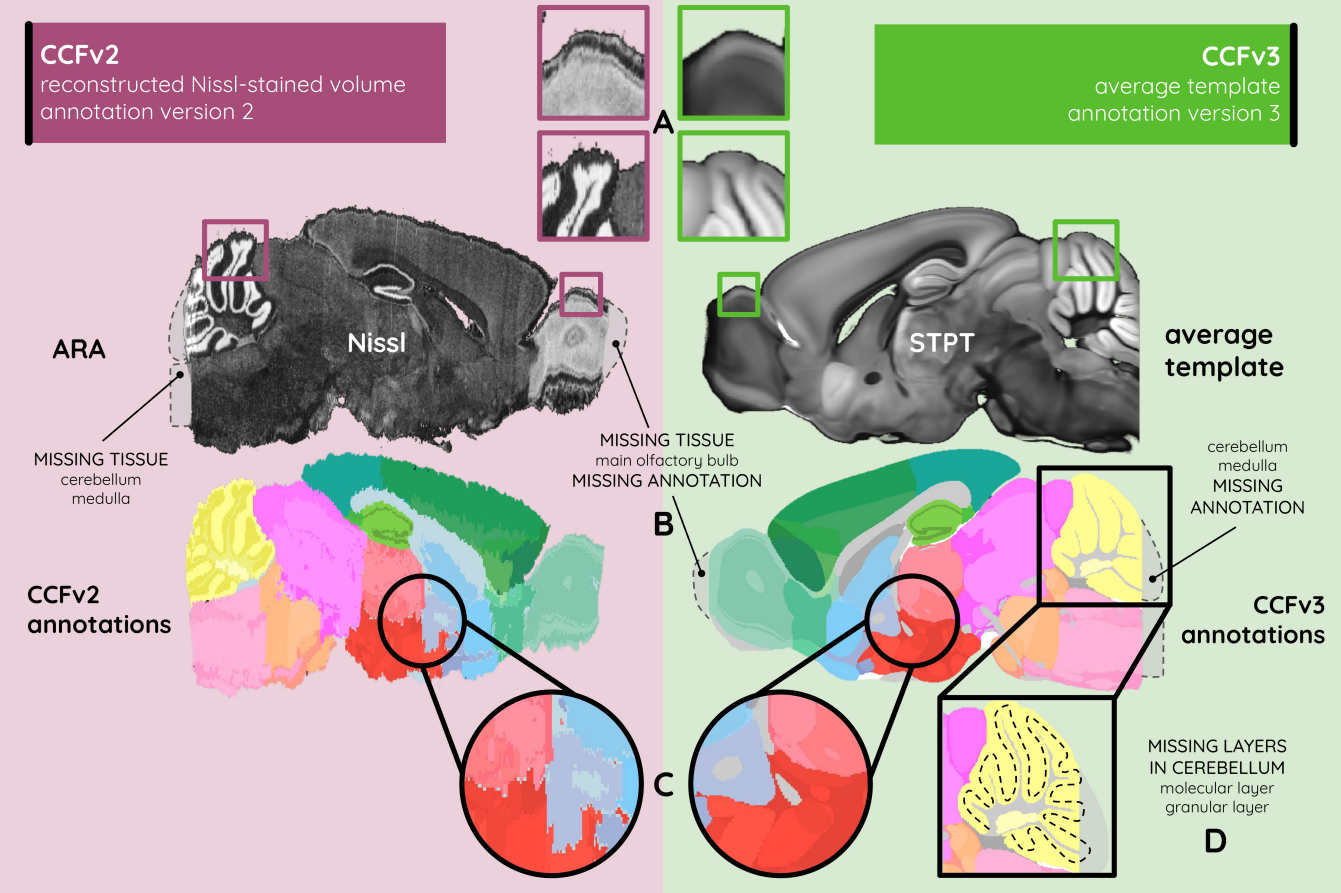
The two latest versions of the atlas provided by the AIBS. The CCFv2 (burgundy) and CCFv3 (green) with their respective characteristics: (A) in contrast, texture, and smoothness of the anatomical tissue; (B) in the missing main olfactory bulb, cerebellum, and medulla tissue and annotation; (C) in the 3D smoothness of the annotation; and (D) in subdivisions from the cerebellar annotation.

The two most recent versions of the AIBS CCF are CCFv2 and CCFv3 (Q. Wang et al., 2020;Fig. 1), each constructed using distinct template volumes. The CCFv2 is based on the reconstructed ARA, while the CCFv3 is built on the STPT average template. Both versions accurately represent mouse brain anatomy but differ in their approaches and strengths. The ARA is highly valuable for the automated registration of both Nissl and*in situ*hybridization (ISH) stained histology since ISH tissue often has an appearance comparable with Nissl. However, the ARA excludes the most rostral and caudal coronal slices of the brain, addressing challenges such as tissue distortion, damage, and artifacts frequently observed in the outermost slices, which could otherwise compromise data accuracy and reliability (Dong, 2007;Xiong et al., 2018).

In contrast, the STPT average template introduces a different set of considerations. While averaging data from multiple brains enhances anatomical representativeness, it also reduces image texture and contrast due to inter-individual variability, making the template less comparable with single-brain data commonly produced by conventional histological platforms (Fig. 1A). Furthermore, the STPT template’s methodology, rarely used in standard laboratory settings, has sparked debate within the scientific community regarding its utility as a reference standard (Rodarie et al., 2022;Stæger et al., 2020). Consequently, researchers must perform multimodal registration between their histological data and the STPT template to utilize CCFv3 for segmentation. Multimodality (or intermodality) refers to the acquisition, integration, and analysis of images obtained from two or more different imaging modalities, each providing different information about the same biological structure or tissue. These modalities may differ in their underlying physical principles, contrast mechanisms, spatial or temporal resolution, or the type of biological information they capture.

The CCFv2 annotation volume offers advantages in its more detailed labeling of gray matter compared with CCFv3. However, CCFv2 annotations were delineated exclusively in the coronal plane, limiting their reliability for sagittal and horizontal analyses (Fig. 1C;Erö et al., 2018;Krepl et al., 2021;Rodarie et al., 2022). The CCFv3 annotations were smoothed and validated in 3D using a large multimodal dataset, significantly improving usability for 3D atlas-based segmentation and visualization in any orientation (Fig. 1C). Both the CCFv2 and the ARA provide incomplete brain representations, omitting structures such as the olfactory bulb, cerebellum, and medulla (Fig. 1B). Within the cerebellum, a key structure comprising 16 lobules (White et al., 2014) organized into three layers (granular, molecular, and Purkinje, the latter being located between the first two), the granular and molecular layers included in CCFv2 are absent in CCFv3 (Fig. 1D).

Single-modality (or monomodality, intramodality) registration, in which the template and the data being registered are of the same type, typically produces higher-quality results compared with multimodal registration, where the template and data differ (Chiaruttini et al., 2022;Goubran et al., 2019). For single-modality registration, the ARA available in CCFv2 is often the preferred anatomical reference, while most researchers rely on the CCFv3 annotations. A version of the ARA aligned to the CCFv3 space would offer a significant advantage, enabling single-modality registration while retaining the benefits of CCFv3 annotations. Despite attempts by the AIBS to improve alignment, the correspondence between the ARA and CCFv3 remains suboptimal, with significant misalignments in regions such as the hippocampus, cerebellum, and others. Additionally, the pre-registered ARA in the CCFv3 space is asymmetrical, despite the symmetry of the original ARA and STPT average template, further limiting its precision. Despite this, the ARA remains frequently used in some studies (Zhang et al., 2023), potentially leading to inaccurate results as reported byRodarie et al. (2022).

The increasing use of 3D digital atlases reflects the growing demand for precise localization of brain data, particularly in fields such as*in silico*modeling (Markram, 2006;Markram et al., 2015). A complete brain atlas, free of the limitations imposed by histological sectioning, is especially valuable for regions like the olfactory bulb (Imamura et al., 2020;Kosaka & Kosaka, 2016;Licht et al., 2023) and cerebellum (Golpinar & Demir, 2020;Kebschull et al., 2024;Pisano et al., 2021). The olfactory bulb is critically involved in Alzheimer’s and Parkinson’s diseases, where olfactory dysfunction often serves as an early indicator of neurodegeneration (Attems et al., 2015;Doty, 2017). It is also affected by SARS-CoV-2, which can cause transient or persistent anosmia through viral invasion and inflammation (Meinhardt et al., 2021). The cerebellum is affected in autism spectrum disorder, spinocerebellar ataxias, and multiple system atrophy, with alterations in Purkinje cells and circuitry (Ashizawa et al., 2018;Fatemi et al., 2012). Improved cerebellar annotations in our new atlas version enhance structural segmentation for studying these disorders. Similarly, the brainstem and medulla are implicated in amyotrophic lateral sclerosis and multiple sclerosis (Haider et al., 2016;Turner et al., 2013).

Although several efforts have been made to address missing brain regions (Bertrand & Nissanov, 2008;Perens et al., 2023;Pisano et al., 2021;N. Wang et al., 2023;Young et al., 2021), no extended version of the CCFv3 encompassing the central nervous system has been created to date. Other groups have constructed atlases based on the CCFv3 using different modalities such as light-sheet microscopy or magnetic resonance imaging (Perens et al., 2023;N. Wang et al., 2023). Although the templates for these other atlases include either the entire olfactory bulb, and/or cerebellum and brainstem, these regions remain unannotated. As a result, researchers studying these areas are often limited to extrapolating from adjacent atlas sections (Zhu et al., 2023), or excluding data that fall outside atlas boundaries entirely (Yuan et al., 2023).

**Fig. 2.**
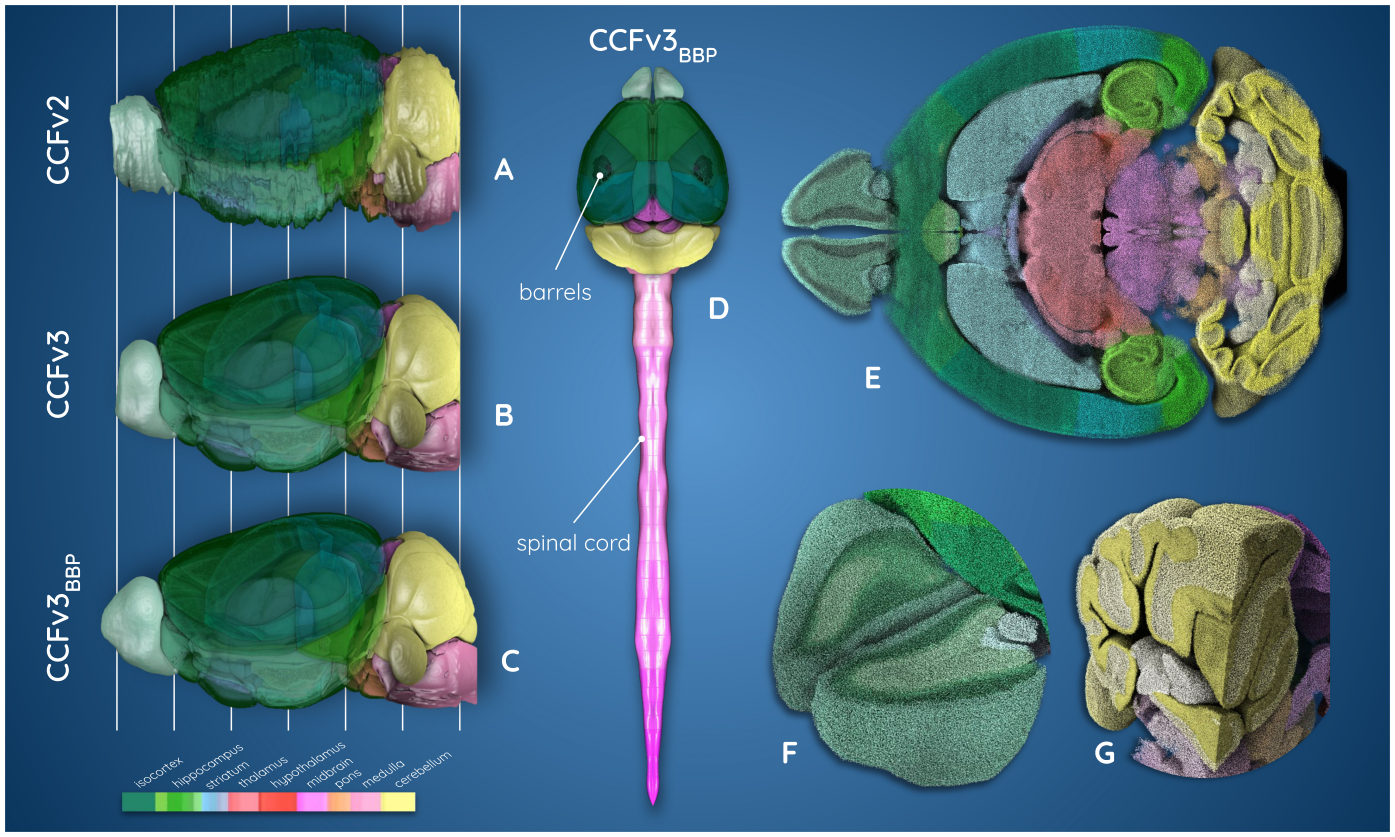
Atlas versions from the AIBS and our extended one (colored according to AIBS annotation color schema). (A) CCFv2 annotation, (B) CCFv3 annotation, (C) our CCFv3_BBP_extended annotation, (D) our CCFv3_BBP_central nervous system model including barrel columns, and (E–G) the new cell distribution placement, with each neuron’s soma represented by a sphere, glial cells are excluded here for visualization purposes (E) sliced horizontally, (F) cross section of the main olfactory bulb, (G) cerebellum and medulla (see Video V1 athttps://zenodo.org/records/15176439).

This study addresses the limitations of existing frameworks by delivering a well-aligned Nissl template and a comprehensive atlas that spans the entire mouse brain and spinal cord, offering a valuable resource for the neuroscience community. We present the CCFv3_BBP_Nissl-based atlas, which extends both the ARA and the CCFv3 annotation volumes into ARA_BBP_and CCFv3_BBP_annotations, ensuring comprehensive coverage of the entire mouse brain and spinal cord (Fig. 2; see Video V1 athttps://zenodo.org/records/15176439). We identified and aligned complementary Nissl-stained datasets to fill missing regions in the olfactory bulb, cerebellum, and medulla (Fig. 2E-F). We incorporated granular and molecular layers into the cerebellum using a region-focused registration pipeline (Fig. 2G). Additionally, we designed an automated and reproducible method for precisely aligning the ARA to the CCFv3 space, transforming multimodal registration into a monomodal problem to achieve detailed 3D alignment. By aligning 734 Nissl-stained mouse brains from the AIBS database (Lein et al., 2007;Ng et al., 2007) within the CCFv3_BBP_framework, we generated an average Nissl template, refined cell distribution placements (Fig. 2E), and integrated recent cortical and spinal cord annotations, resulting in a fully annotated mouse central nervous system (Fig. 2D).

While the AIBS and Waxholm mouse brain atlases have enabled large-scale brain-wide analyses (Leergaard & Bjaalie, 2022), our new atlas extends this capability to the central nervous system. By enabling precise alignment of histologically stained data from other laboratories to the CCFv3_BBP_through registration with the aligned ARA_BBP_, our atlas addresses a major limitation of previous frameworks and enables the comparison of a wide variety of histological staining within a unified reference space. This extended atlas version, which covers the entire brain, significantly broadens its applications, particularly for researchers focusing on the main olfactory bulb, cerebellum, and brainstem. To illustrate its utility, we provide two key applications where the extended and aligned ARA_BBP_enabled significant advancements in mouse brain data segmentation using 3D digital atlases. First, we constructed a 3D average Nissl template from thousands of histological slices. Second, we refined cell distribution and identification across the entire mouse brain.

## 2 Material and Methods

### 2.1 Source data

The source data used in this study come from the Allen Institute for Brain Science (AIBS) and can directly be downloaded from its platform (seeSupplementary Material S1-S2). The two latest versions are (1) CCFv2 and (2) CCFv3 (Allen Institute for Brain Science, 2015;Q. Wang et al., 2020). Both versions are available in isotropic resolutions of 25 µm and 10 µm. The original structural annotations are organized hierarchically, and the hierarchy file is the same for both versions. Both CCFv2 and CCFv3 annotation datasets have their own number of anatomical regions, which is less than the total number of regions present in the hierarchy. This number varies for each version depending on the experimental data used to produce the annotation file.

To reconstruct the missing parts of the ARA reference, we used a sagittally sectioned whole mouse brain from the AIBS (NisslSAG, Allen Mouse Brain Atlas ID 100042147;Allen Institute for Brain Science, 2011;Kuan et al., 2015). It was downloaded at both isotropic resolutions of 25 μm and 10 μm (seeSupplementary Material S1-S2for more details). We also made use of another whole mouse brain from the Waxholm space sectioned in the horizontal incidence (NisslHOR) that we reconstructed in 3D (seeSupplementary Material S1-S2for more details) with a raw resolution of 9.9 ☓ 21.0 ☓ 9.9 µm^3^and resampled to an isotropic resolution of 21 µm (G. A. Johnson et al., 2010). All data correspond to adult C57BL/6J mouse brains.

### 2.2 Methods

**Fig. 3.**
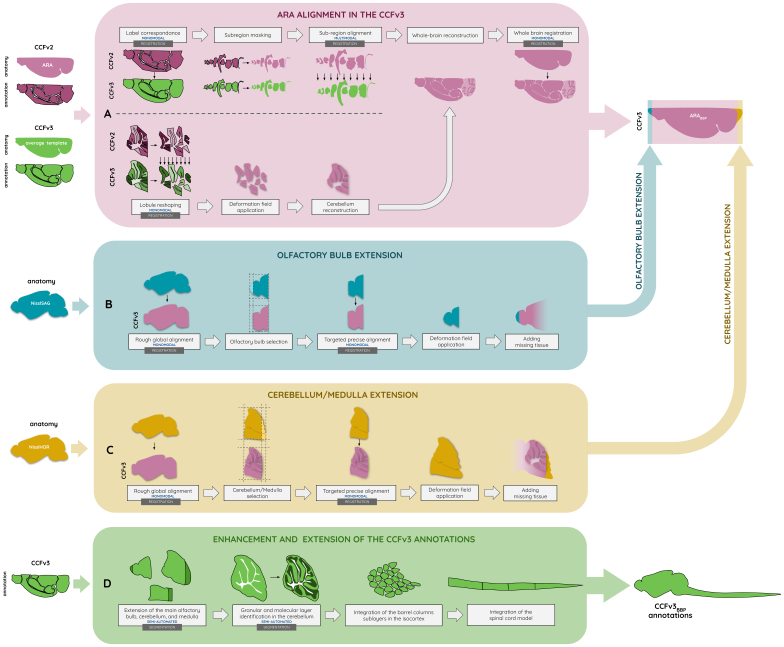
Flowchart for producing the CCFv3_BBP_atlas, with the automated registration pipeline for producing the ARA_BBP_in (A) aligning the ARA in the CCFv3 using a dedicated method for the cerebellum based on the CCFv2 and CCFv3 atlases, (B) extending the main olfactory bulb using the NisslSAG data, and (C) extending the cerebellum and medulla using the NisslHOR data, as well as (D) the production of the enhanced and extended CCFv3_BBP_annotations (seeSupplementary Material S4for more details).

To build an improved annotation and Nissl-based atlas, we first ensured data compatibility through preprocessing steps before applying a registration pipeline. This pipeline (Fig. 3) consists in a set of three automated registration methods for aligning the ARA in the CCFv3 and extending it to the entire brain, alongside two semi-automated methods for identifying the cerebellar sublayers and incorporating the missing layers covering the extended regions of the ARA_BBP_. To build the ARA_BBP_, the ARA was first aligned to the CCFv3 (Fig. 3A), next the rostral part of the brain was extended (Fig. 3B), and finally, the caudal extension was incorporated (Fig. 3C). All the Nissl tissue corresponding to the extension added from the NisslSAG and the NisslHOR was merged into the ARA reference volume, in accordance with the AIBS initiative, maintaining a consistent common reference framework. Furthermore, the NisslSAG and the NisslHOR volumes were independently aligned to the final ARA_BBP_, generating NisslSAG_BBP_and NisslHOR_BBP_, to integrate them as additional data into the CCFv3_BBP_framework.

The objective of image registration is to find the optimal spatial transformation that aligns the*moving*image, on which the transformation is estimated and applied, with the*fixed*image (reference), while ensuring that corresponding points in both images match as accurately as possible (Glasser et al., 2016). For image registration, we made use of freely available state-of-the-art algorithms, Advanced Normalisation Tools (ANTs;Avants et al., 2008) and NiftyReg (Modat et al., 2014), both of which have demonstrated high efficacy in registering mono- and multimodal biomedical 2D and 3D datasets across various applications (Balakrishnan et al., 2019;Borovec et al., 2020;Iglesias et al., 2023;Mano et al., 2020;Murakami et al., 2018;Nazib et al., 2018;Niedworok et al., 2016). The ANTs algorithm, particularly its*symmetric image normalization*(SyN) transformation (affine and deformable transformation, with mutual information as optimization metric), is well suited for the nonlinear alignment of small datasets, such as 2D histological slices (Goubran et al., 2019;Krepl et al., 2021). However, its performance declines when applied to large high-resolution volumes (Johnson et al., 2023). Conversely, NiftyRegF3D, the nonlinear image registration algorithm part of NiftyReg and based on a block-matching approach, free-form deformation, and normalized mutual information (NMI) similarity metric, is highly effective for multimodal registration tasks (Modat et al., 2014). We also used the visualization and segmentation software ITK-snap (Yushkevich et al., 2006) for assessing the resulting images.

To illustrate the utility of this new atlas version, we present two key use cases. The first involves generating a population-averaged Nissl template within the CCFv3_BBP_space, providing a robust reference for comparative neuroanatomy. The second focuses on precisely aligning 2D gene expression histological slices within the 3D atlas, enabling a more accurate estimation of cellular density for different cell types across the entire mouse brain. This effort contributes directly to the BBP cellular atlas initiative, which aims to construct and simulate an*in silico*model of the complete mouse brain (Rodarie et al., 2022). Finally, we provide comprehensive guidelines on data accessibility, downloading procedures, and usage instructions for integrating this atlas into neuroanatomical and computational neuroscience research.

### 2.3 Development of the CCFv3 _BBP_ atlas

#### 2.3.1 Preprocessing

The NisslHOR dataset was resampled to an isotropic resolution of 25 µm and reconstructed in 3D using an adjacent rigid slice-to-slice registration technique, which preserves straight-line transformations, following the method proposed by prior research (Ourselin et al., 2001). To ensure consistency with this resolution, the latest CCFv3 annotation volume from the AIBS platform was also downsampled to 25 µm using an in-house algorithm (seeSupplementary Material S3.1). Furthermore, isolated and discontinuous voxels located at the extreme borders of the CCFv3 annotation volume, beyond the brain annotations, were removed to reduce discontinuities and jagged artifacts (seeSupplementary Material S3.2).

The CCFv3 annotations are not symmetrical according to the inter-hemispheric plane; part of the dor*s*al tegmental decussation region, including 433 voxels, is present on the left hemisphere only (seeSupplementary Material S3.2). As the data are assumed to be symmetric along this plane, processing was conducted on a single hemisphere, which was subsequently duplicated to restore bilateral symmetry. The right hemisphere was selected for this procedure, as its CCFv3 annotations align more accurately with the ARA (Krepl et al., 2021;Rodarie et al., 2022). Additionally, a portion of the main olfactory bulb was approximated by inheriting the glomerular layer from its child region, as this layer was absent in the latest CCFv3 annotation version (seeSupplementary Material S3.2).

All methods were implemented at a 25 µm isotropic voxel resolution. The estimated deformation fields were subsequently upsampled to 10 µm resolution and applied to the raw Nissl-stained data at the same resolution. The final extended annotation, incorporating the granular and molecular layers across all cerebellar lobules, was upsampled to 10 µm isotropic resolution and smoothed to enhance spatial continuity.

#### 2.3.2 Precise alignment of the ARA in the CCFv3

To achieve a precise alignment of the ARA within the CCFv3, we developed an automated registration method that ensured regional accuracy while considering the anatomical characteristics of the entire brain (Fig. 3A). A key challenge was the large-scale multimodality inherent in the dataset, which required an approach prioritizing region-by-region registration rather than a single global nonlinear transformation. This approach made it possible to bypass the need for a global nonlinear transformation, which involves complex deformations to achieve precise alignment, and instead prioritized a series of localized sub-transformations tailored to each region (seeSupplementary Material S4for more details).

A fundamental aspect of this alignment method was the reciprocal combination of anatomical and annotation files from the CCF, which enabled accurate region selection. However, although both annotations rely on the same hierarchy file, some regions exist in the CCFv2 annotations that are not included in the CCFv3 annotations, and vice versa (seeSupplementary Material S5). As those disparities are notably pronounced in the white matter regions, making them difficult to compare, our registration focused on gray matter regions. Specifically, the CCFv3 annotations exhibit a 14% reduction in the number of gray matter regions compared with CCFv2 annotations. As a result, only 527 leaf regions are directly comparable between the two versions. For the rest of the regions, we considered another parent level from the hierarchy.

Two ontology levels were defined: the*leaf*level, which represents the deepest common hierarchical regions between the two annotation versions, and the*parent*level, representing an intermediate hierarchy when common leaf labels were absent (seeSupplementary Material S5). The parent level was determined based on the first shared anatomical structure between CCFv2 and CCFv3, regions for which a non-zero set of voxels is defined with the same label in both annotation volumes, ensuring that registration preserved the hierarchical integrity of the annotations. This hierarchical approach allowed for high-precision alignment at the leaf level, while concurrently addressing gaps in annotation differences at the parent level.

Alignment at the two different levels (leaf and parent) was performed independently. The registration process began with an initial monomodal linear (including translation, rotation, scaling, and shearing) and nonlinear alignment (SyN transformation) between CCFv2 annotations (moving) and CCFv3 annotations (fixed), using ANTspy (Krepl et al., 2021) to generate a transformation that was subsequently applied to the ARA with linear interpolation. Following this, the ARA was subdivided into multiple subregions using CCFv2 annotation masks at both leaf and parent ontology levels, and the STPT average template was segmented similarly with CCFv3 annotation masks. Each of these masked subregions was independently registered using a linear transformation, followed by a nonlinear multimodal registration (NiftyRegF3D), aligning the ARA (moving) to the STPT average template (fixed). Once each region was precisely registered, the whole-brain tissue was reconstructed by merging all registered masked voxels from the leaf ontology level while filling any missing tissue using data from the parent ontology level. A final monomodal nonlinear whole-brain registration was performed between the raw ARA (moving) and the reconstructed ARA (fixed) to preserve the integrity of brain tissue and minimize unrealistic deformations.

Due to the lack of sublayers in the cerebellum, standard registration methods were insufficient at the lobular scale. The high contrast between granular and molecular layers, which is absent in the STPT average template, required a dedicated cerebellar alignment strategy that first identified the granular and molecular labels in the CCFv3 (Fig. 3A). To address this, each of the 16 masked cerebellar lobules was independently registered between CCFv2 annotation (moving) and CCFv3 annotation (fixed) using an affine transformation, followed by an*aggressive symmetric normalization*(SyNAggro) algorithm in ANTs, which included fine-scale matching and enhanced deformation, coupled to the*demons*similarity metric (Avants et al., 2008;Modat et al., 2014). The resulting 3D transformations were applied to align the 16 masked lobules from the ARA within the CCFv3 framework. Once registered, they were merged into the reconstructed whole ARA_BBP_volume to complete the Nissl-stained representation of the cerebellum before the final monomodal nonlinear whole-brain registration step.

To evaluate the accuracy of the registration, we used the multimodal NMI similarity metric between the ARA and the STPT average template (Equation 1). NMI was computed at multiple levels: at the whole-brain scale after the final step, at the level of each registered subregion to ensure the homogeneity of the registering improvement across all subregions, and at the level of individual slices, considering the volume as a succession of slices in the three conventional incidences (coronal, sagittal, and horizontal). This multi-scale analysis ensured a comprehensive evaluation of whether the similarity between the two anatomical volumes improved after registration. NMI is defined such as

$$\text{NMI}\left(A,B\right)=\frac{2\times I\left(A;\;B\right)}{H\left(A\right)+H\left(B\right)},$$ (Equation 1)

where*I*(*A*;*B*) is the mutual information between images*A*and*B*,*H*(*A*) is the entropy of image*A*, and*H*(*B*) is the entropy of image*B*(Studholme et al., 1998).

Additionally, we used an independent metric to assess whether the resulting aligned ARA_BBP_was better fitting the CCFv3 annotations based on 29 fiducial anatomical points of interest, carefully selected for their clear identification in the tissue and their distribution across the brain (Bjerke et al., 2018). These points were selected because they are distributed throughout the entire brain and are located at the boundaries between different regions (seeSupplementary Material S7.1). These points spanned seven major brain regions: the olfactory areas, cerebellum, hippocampus, striatum, brainstem, thalamus, and cortex. Given this set of reference points readily identifiable, five operators with diverse backgrounds were tasked with identifying these points before (ARA) and after (ARA_BBP_) registration (seeFig. 6K). We evaluated and compared the target registration error (TRE) in calculating the Euclidean distance between each point defined by an operator and the reference set of points in the CCFv3 annotations both before and after registration in the 3D volume (Equation 2), defined as

$$\text{TRE}=\sqrt{{\left(x_{\text{ref}}-x_{\text{reg}}\right)}^{2}+{\left(y_{\text{ref}}-y_{\text{reg}}\right)}^{2}+{\left(z_{\text{ref}}-z_{\text{reg}}\right)}^{2},}$$ (Equation 2)

where*x*_ref_,*y*_ref_, and*z*_ref_are the coordinates of the fiducial point in the reference image (CCFv3 annotations), and*x*_reg_,*y*_reg_, and*z*_reg_are the coordinates estimated in the ARA before and ARA_BBP_after registration (Bookstein, 1989).

Codes for this automated registration method embed a high-performance computing infrastructure for decreasing the computation time. This method was run using Blue Brain 5, which is a heterogeneous supercomputing cluster with resources adapted to the scientific requirements of the project. In particular, we used 40 nodes from the cluster in which each node features 2 Intel Xeon Gold 6248 CPUs (*i.e.*, 40 core per node), 384 GB of memory, and are interconnected through Infiniband EDR. In total, our evaluations utilized 1600 processes distributed using the multiprocessing Python library (https://docs.python.org/3.13/library/multiprocessing.html).

#### 2.3.3 Rostral and caudal extension of the ARA _BBP_

To extend the ARA_BBP_reference, additional tissue from the NisslSAG and NisslHOR datasets was incorporated, allowing for the completion of the main olfactory bulb, cerebellum, and medulla. These datasets, being Nissl stained, were well suited for monomodal registration, ensuring consistency in tissue contrast and structural alignment. Since the entire main olfactory bulb is present in the NisslSAG, this dataset was used to reconstruct and integrate the missing rostral regions of the ARA_BBP_. To accommodate the dimensions of the NisslSAG, the borders of the ARA_BBP_were padded with zero values, ensuring proper spatial alignment.

The process of integrating the rostral extension involved a sequence of registration and transformation steps (Fig. 3B). First, the entire NisslSAG dataset (moving) was linearly registered in 3D to the ARA_BBP_(fixed) using the ANTs Affine mode (rigid and scaling). Once aligned, both datasets were cropped to isolate the olfactory bulb region using a bounding box [MOB] from the CCFv3 annotations, producing ARA_BBP_[MOB] and NisslSAG[MOB]. To ensure tissue correspondence, the NisslSAG[MOB] was masked using truncated CCFv3 annotations to hide the missing part of the olfactory bulb and match the ARA_BBP_[MOB] tissue covering. This masked NisslSAG[MOB] (moving) was then applied nonlinear registration using ANTs SyNOnly mode (SyN with no rigid or affine stages) with ARA_BBP_[MOB] (fixed), yielding a transformation matrix. The computed transformation was applied to the entire unmasked NisslSAG[MOB], ensuring that all voxels including the tissue extension were correctly mapped to the ARA_BBP_space with continuity. Histogram matching normalization was performed between the aligned NisslSAG[MOB] and ARA_BBP_[MOB], minimizing intensity differences between datasets. The missing voxels in the ARA_BBP_main olfactory bulb were then added to it using the aligned Nissl_SAG_[MOB]. To ensure a smooth transition between the two brain parts at the junction between ARA_BBP_and the added tissue from NisslSAG, voxel intensities were averaged along a three-voxel-thick boundary along the rostro-caudal axis. The final main olfactory bulb extension was assessed by experts to ensure anatomical consistency.

For the caudal extension, the NisslHOR dataset was used, as it contains the entire cerebellum and medulla, which were missing in the original ARA. The four most caudal lobules were specifically concerned by the extension: the folium-tuber vermis (VII), uvula (IX), paramedian lobule, and copula pyramidis. The same registration and transformation procedure applied to the olfactory bulb was followed for the cerebellum and medulla (Fig. 3C). A bounding box encompassing these regions was defined in both the NisslHOR and ARA_BBP_, followed by linear registration, cropping, masking using CCFv3 annotations, nonlinear SyNOnly registration, transformation application, histogram matching, and junction smoothing. The final cerebellum and medulla extensions were assessed by experts to ensure anatomical consistency.

#### 2.3.4 Integration of new anatomical labels in the CCFv3 _BBP_

To ensure precise alignment of the ARA_BBP_within the CCFv3, it was first necessary to identify and incorporate the missing granular and molecular layers across all cerebellar lobules in the CCFv3 annotations (Fig. 3A and D). Sixteen cerebellar lobules were involved in this sub-segmentation: the lingula (I), lobules (II–V), declive (VI), folium-tuber vermis (VII), pyramus (VIII), uvula (IX), nodulus (X), simple lobule, crus 1-2, paramedian lobule, copula pyramidis, paraflocculus, and flocculus.

A semi-automated segmentation method was developed to delineate the granular and molecular layers in the CCFv3 based on Nissl-stained contrast differences from the aligned ARA_BBP_(Fig. 3D; seeSupplementary Material S9; seeSupplementary Material S3.4). This approach used binary Otsu thresholding (Otsu, 1975), an image processing technique effective in automatically separating the dense granular layer from the rest of the lobule. We applied this technique independently to each of the 16 cerebellar lobules. The granular layer was assigned to the highest intensity voxels, corresponding to high cell densities, while the molecular layer was mapped to low-intensity regions, reflecting lower cell densities, in accordance with the 32 leaf regions defined in the hierarchy file (Hayashi et al., 2017;Šišková et al., 2013).

Given that Purkinje cells exhibit a soma diameter ranging from 20 to 25 µm (J. Kim et al., 2011) and form a single-layer structure at the interface of the granular and molecular layers of each cerebellar lobule (Šišková et al., 2013), a 25 µm voxel resolution was insufficient to accurately delineate the thin layers and may cause significant discontinuities. However, since the CCFv3 annotation was also available at 10 µm resolution, a thin two-voxel layer representing the Purkinje layer was automatically generated along the boundary between the granular and molecular layers, once they were upsampled at that resolution. This approach provided an effective balance between anatomical accuracy and sampling constraints.

Another semi-automated labeling method was applied to the newly reconstructed tissue from the ARA_BBP_, incorporating image processing techniques with minimal manual corrections (Fig. 3D; seeSupplementary Material S3.4). Following the digital reconstruction of the olfactory bulb, cerebellum, and medulla, this approach was employed to identify new layers in continuity with those defined in the CCFv3 annotations to produce the CCFv3_BBP_annotations. The rostral extension concerns five layers in the main olfactory bulb (granular, inner plexiform, mitral, outer plexiform, and glomerular layers), while the caudal extension concerns eight layers in the cerebellum (granular and molecular layers for the folium-tuber vermis (VII), uvula (IX), paramedian lobule, and copula pyramidis), plus one additional layer for the medulla. Furthermore, the arbor vitae region from the fiber tracts was slightly manually extended in the uvula (IX) lobule to align with the expanded brain regions.

Building upon the ARA_BBP_anatomical contrast, experts manually delineated the extended tissue regions as well as the granular and molecular layers in all cerebellar lobules, producing a labeled version of all regions added in the CCFv3_BBP_annotations (Fig. 3D; seeSupplementary Material S3.3;Colnot et al., 2025;L’Yvonnet et al., 2025). This process was further supported by the Paxinos and Franklin atlas fifth edition (Paxinos & Franklin, 2019) as an additional atlas reference, alongside expert-delineated NisslSAG annotations on the web interface from the AIBS (Allen Institute for Brain Science, 2011).

To assess the result from the semi-automated methods, the CCFv3_BBP_newly added annotations were compared with the expert-generated annotation file, the latter being used as a reference. A visual assessment was performed in three standard orientations using ITK-Snap visualization software, and a Dice similarity coefficient (Dice, 1945) was calculated for each label, taking expert annotations as the reference (A) and the CCFv3_BBP_annotations as the target (B). The Dice score (Equation 3) was computed as

$$\text{Dice score}=\frac{2\times \left|A\cap B\right|}{\left|A\right|+\left|B\right|},$$ (Equation 3)

where |A⋂B| is the number of voxels that are common between the reference and target labels, |A| the number of voxels in the reference label A, and |B| the number of voxels in the target label B. This score ranges from 0 (no overlap) to 1 (perfect overlap).

In addition to these label refinements and additions, further enhancements were made by incorporating newly available anatomical annotations. The barrel column annotation for the somatosensory cortex, developed in recent studies (Bolaños-Puchet et al., 2024), as well as the spinal cord model (Kuras, 2023), were merged into the CCFv3_BBP_annotations, resulting in a more complete representation of the mouse central nervous system (Fig. 3D). A combination of volume concatenation and merging techniques was used to integrate these new datasets, ensuring a coherent and more comprehensive extended version of the atlas.

### 2.4 Applications and data accessibility

#### 2.4.1 Generation of a population-averaged Nissl of the entire mouse brain

**Fig. 4.**
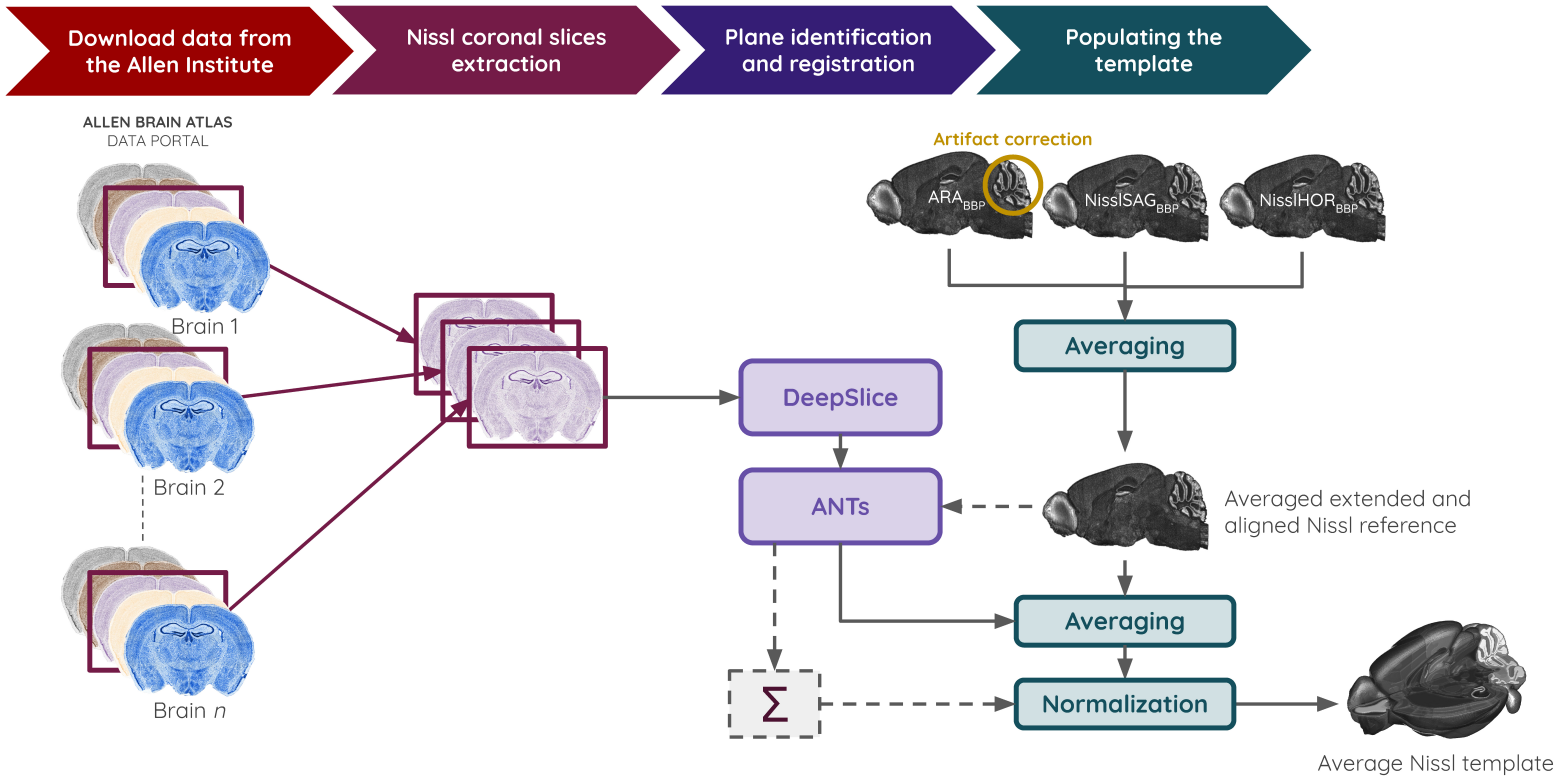
Automated pipeline for generating the population-averaged Nissl template of the entire mouse brain from the new ARA_BBP_data, NisslSAG_BBP_, NisslHOR_BBP_, and all coronal Nissl slices from the AIBS*in situ*hybridization data portal.

While a single Nissl-stained reference atlas, constructed from complementary brains (ARA_BBP_, NisslSAG_BBP_, and NisslHOR_BBP_), is well suited for registration purposes, certain applications benefit from a population-averaged Nissl template derived from multiple specimens. For example, when Nissl intensity is used as an indicator for cell density (Erö et al., 2018;Rodarie et al., 2022), an averaged dataset incorporating multiple individuals provides a more representative and statistically robust estimation. Building on the CCFv3_BBP_, we aligned 734 Nissl-stained mouse brains to the ARA_BBP_, generating an average Nissl-stained template within this standardized framework (Fig. 4).

We first initialized the template with the average of the ARA_BBP_, NisslSAG_BBP_, and NisslHOR_BBP_, all aligned in the CCFv3_BBP_framework. We took this volume as a reference (fixed) for all the alignment process. Before merging the volumes, we applied an automated method based on outliers identification and cell density rules to correct significant tear artifacts in the ARA_BBP_cerebellum (seeSupplementary Material S9.2A). To build this population-based template, we downloaded 86,901 coronal Nissl-stained slices from 734 post-natal day 56 C57BL/6J mouse brains from the AIBS*in situ*hybridization data portal (Lein et al., 2007;Ng et al., 2007). The DeepSlice registration solution (Carey et al., 2023) was used to approximate the initial position of each section within the CCFv3_BBP_. Although AIBS provides sagittal sections, they were excluded from the analysis as they cover only one hemisphere, and DeepSlice is optimized exclusively for coronal slices. Following automated registration, each dataset underwent manual expert inspection and correction to refine angle and position alignment, with only minor adjustments required.

Once pre-aligned, the Nissl sections were nonlinearly registered to their corresponding anatomical planes in the initialized average Nissl volume, using the SyN transformation from the ANTspy registration library. The voxel intensities from each section were then mapped onto a 10 µm isotropic resolution volume within the CCFv3_BBP_. This process was repeated across all Nissl datasets, summing voxel intensities where overlapping sections from different specimens intersected.

Since each Nissl dataset only covered a subset of the voxels, the final volume initially contained variable voxel intensity distributions depending on the number of overlapping sections. To correct for this, we calculated the number of sections contributing to each voxel and subsequently normalized the values by dividing each voxel intensity by the total number of sections intersecting it (in addition to the average Nissl initialized reference). The final output was a population-averaged Nissl volume, where each voxel accurately reflected the mean Nissl intensity derived from all intersecting datasets, providing a consistent and anatomically representative template. Using the ARA_BBP_as first reference for these data ensured that every voxel from the average Nissl template has an intensity value in the CCFv3_BBP_framework.

#### 2.4.2 Spatial alignment of gene expression data for the BBP cellular atlas

**Fig. 5.**
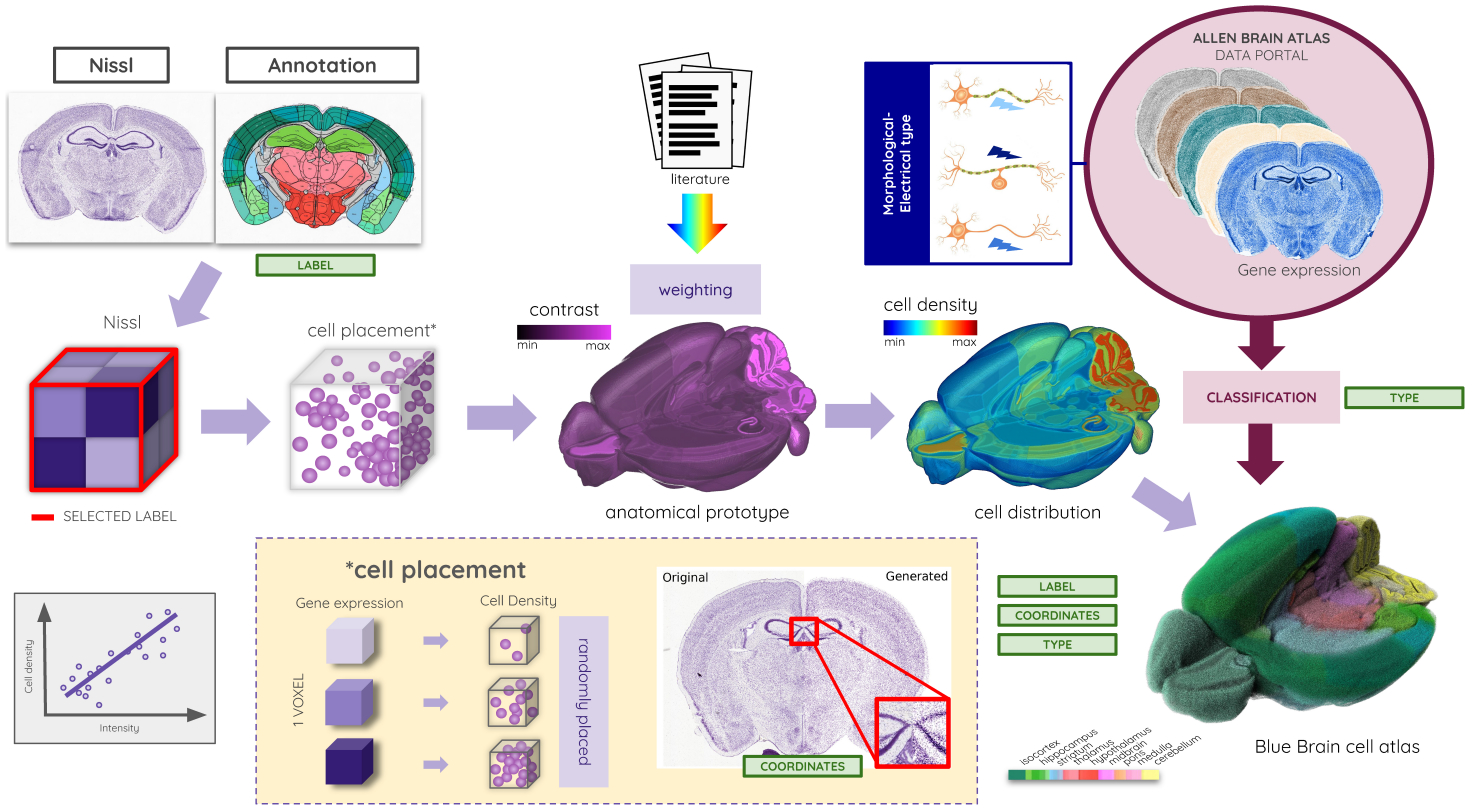
The Blue Brain Cell Atlas model generated from the new CCFv3_BBP_atlas version and gene expression from the AIBS*in situ*hybridization data portal, providing the label, coordinates, and type for all cells in the entire mouse brain.

The Blue Brain Cell Atlas, produced by the Blue Brain Atlas pipeline, is the first digital 3D cell atlas of the mouse brain, offering neuroscientists an unprecedented view of its cellular composition. It provides detailed data on the densities and 3D coordinates of all cells across the brain regions defined by the Allen Mouse Brain Atlas (Erö et al., 2018;Rodarie et al., 2022). Specifically, the cell atlas pipeline identifies excitatory, inhibitory, and neuromodulatory neurons, as well as astrocytes, oligodendrocytes, and microglia, enabling comprehensive analyses of brain structure at the cellular level. The atlas was constructed by integrating the ARA and gene expression stains from the AIBS, where gene expression patterns were used to classify major cell types. From these cell types, distinct morphological–electrical subtypes were derived, contributing to the construction of a comprehensive*in silico*model of the mouse brain. The number of cells and spatial distributions were validated against literature-reported values to ensure consistency. This pipeline generated a detailed cell distribution, providing the label, coordinates, and type of every cell in the mouse brain (Fig. 5).

This cell atlas was constructed by integrating features from both CCFv2 and CCFv3 annotations, leveraging the advantages of each: the greater number of defined brain regions in CCFv2 and the improved smoothness of anatomical structures in CCFv3. The final atlas was designed to be fully aligned with the CCFv3 framework. However, the initial version had two major limitations: it did not cover the entire mouse brain, and its cell distribution relied solely on the ARA, which was misaligned with the CCFv3. As a result, cell density calculations were performed using CCFv2 annotations, and a transplantation process was applied to approximate densities in CCFv3 by averaging values across comparable regions. This region-based interpolation significantly reduced the spatial precision of the atlas.

By implementing the new CCFv3_BBP_in the BBP Cell Atlas pipeline, these issues were fully addressed. The atlas was extended to cover the entire brain, and the ARA_BBP_was precisely aligned within the CCFv3, eliminating the need for transplantation-based conversion. This enhancement enabled cell density calculations at the voxel level, rather than averaging across anatomical regions, greatly improving the spatial resolution and accuracy of the dataset. Additionally, the removal of the transplantation step streamlined the cell distribution pipeline, reducing computational complexity and decreasing processing time, while further refining the accuracy of the cell distribution model. Utilizing the DeepAtlas suite (https://github.com/BlueBrain/Deep-Atlas), we registered specific markers from the AIBS*in situ*hybridization coronal datasets to our extended CCFv3_BBP_atlas, leveraging the precise alignment provided by the ARA_BBP_. The obtained cell densities in the main regions were compared with state-of-the-art reference values for validation.

Furthermore, the new CCFv3_BBP_atlas enabled the precise alignment of transcriptomic histological data called ABS atlas from the AIBS (Yao et al., 2023) within the CCFv3 framework, a previously unachieved milestone that significantly refines cell classification within the Blue Brain Cell Atlas (Verasztó et al., 2024).

#### 2.4.3 Data and code availability, download, and usage guidelines

All data produced in this study are freely available for open-access download at the following repository:https://zenodo.org/records/15176439. This dataset includes the CCFv3_BBP_, which comprises the ARA_BBP_along with its detailed annotations, as well as the population-averaged Nissl template, the NisslSAG_BBP_, the NisslHOR_BBP_, and additional supplementary data. A comprehensive description of the provided datasets is available directly within the repository. This corresponds to the detailed nomenclature outlined inSupplementary Material S1, ensuring clear guidance for researchers utilizing these resources.

To reproduce all the automated registration methods described in the first part of the Methods section, one can access the dedicated GitHub repository athttps://github.com/BlueBrain/ccfv3a-extended-atlas. The repository provides comprehensive guidelines, including detailed schematics and step-by-step instructions corresponding to theSupplementary Material S4. Additionally, the repository includes scripts to facilitate replication and adaptation for different research needs, making it an essential resource for users looking to apply or extend these methods in their own studies.

The new atlas version has been integrated into the updated Blue Brain Atlas pipeline and is readily available for spatial alignment of gene expression data via the dedicated GitHub repository (https://github.com/BlueBrain/bbp-atlas-pipeline). The Blue Brain Atlas pipeline can also be rerun from this repository to obtain cell densities and their subtypes across the entire mouse brain, along with validation against reported densities in the literature.

The new atlas version was uploaded on the Human Brain Project platform to benefit from the QUINT or the Locare workflows (Blixhavn et al., 2024;Yates et al., 2019). The QUINT workflow (https://quint-workflow.readthedocs.io/en/latest/index.html) is a powerful and user-friendly tool designed for quantifying and analyzing labeled features within a known atlas space, making it particularly well suited for exploring our new CCFv3_BBP_atlas. This workflow facilitates the visualization and analysis of large-scale histological datasets, providing quantitative measurements across atlas-defined regions. Researchers can customize the granularity of regions of interest, tailoring analyses to specific experimental needs. The QUINT suite includes intuitive software tools with graphical user interfaces, eliminating the need for coding expertise. It generates detailed object counts and percentage coverage per brain region, along with 3D point clouds for visualizing spatial distributions of features. By integrating QUINT with the CCFv3_BBP_atlas, researchers gain an efficient and precise framework for mapping, quantifying, and visualizing cellular and molecular features, enhancing the exploration of this newly extended atlas version.

The atlas we developed has also been integrated into the BrainGlobe platform (Claudi et al., 2020), enhancing its accessibility and utility for the neuroscience community (https://brainglobe.info/index.html). BrainGlobe offers a comprehensive suite of Python-based tools designed for computational neuroanatomy, making it an ideal platform for exploring and utilizing our mouse brain atlas. The BrainGlobe Atlas API is ideally designed for accessing, processing, and analyzing the atlas data we provide, offering a unified interface that simplifies dataset integration and programmatic manipulation. By standardizing data formats and access methods, the API ensures seamless interoperability across multiple tools and atlases, significantly streamlining neuroanatomical analysis pipelines. The BrainGlobe platform further enhances atlas exploration through brainrender (Claudi et al., 2021), a powerful Python-based tool for generating high-quality 3D renderings of neuroanatomical structures, enabling intuitive visualization of complex brain architecture. Additional tools are provided for detecting and analyzing cells in large 3D datasets, facilitating efficient data quantification and comparison across studies. With our mouse brain atlas fully integrated into BrainGlobe, researchers can leverage a robust, user-friendly environment that combines standardized data access, advanced visualization, and automated analysis, making it the ideal platform for exploring and utilizing comprehensive brain atlases.

## 3 Results

The ARA was aligned in the CCFv3 and extended using the NisslSAG for the main olfactory bulb and the NisslHOR for the cerebellum and the medulla. We demonstrate in this section how the ARA_BBP_is better aligned in the CCFv3 than the original. Furthermore, we present the new extended CCFv3_BBP_annotation version incorporating the granular and molecular layers in each lobule of the cerebellum plus all extended regions. We also provide an augmented annotation covering the central nervous system directly using the CCFv3_BBP_annotations we produced. Finally, we present results from two key applications of the updated atlas: the production of the average Nissl template and the enhancement of the Blue Brain Atlas.

### 3.1 Alignment and extension of the ARA in the CCFv3

**Fig. 6.**
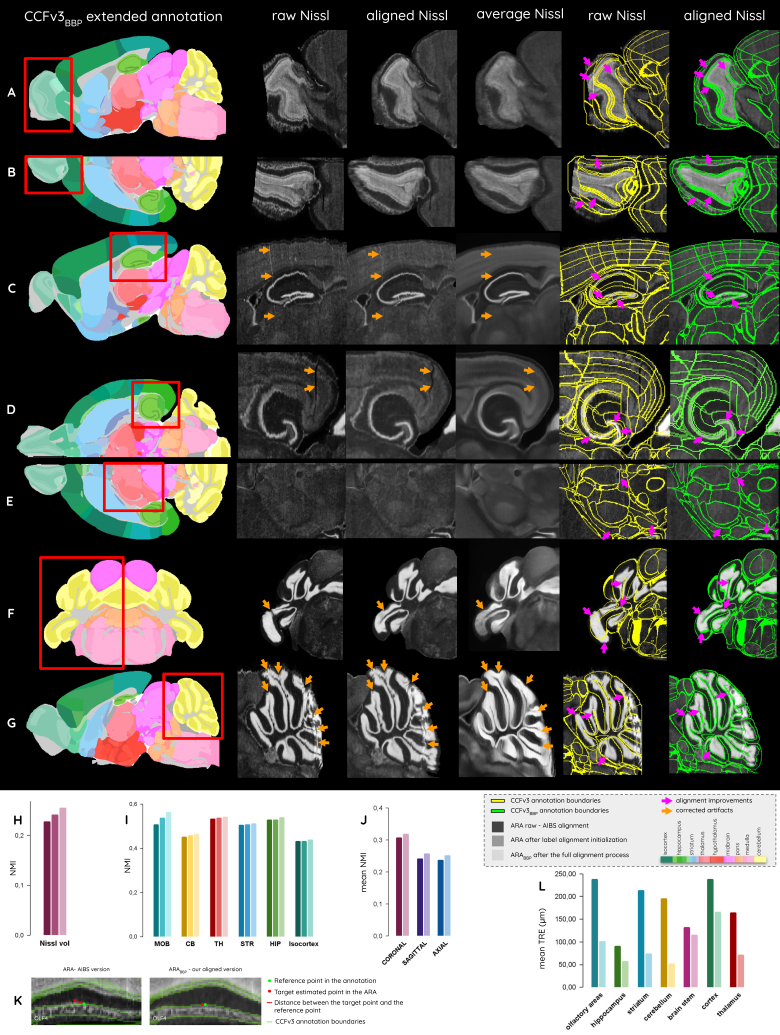
Aligning and extending the ARA in the CCFv3. (A–G) Comparison of the ARA (raw) and ARA_BBP_(aligned) views in the CCFv3, as well as the CCFv3 raw (yellow) and extended CCFv3_BBP_(green) annotation boundaries; (H–J) NMI between the ARA (AIBS alignment, after label alignment initialization, and after the full alignment process) and the STPT average template (H) at whole-brain scale, (I) for 6 main groups of regions, and (J) for all slices in the three conventional incidences. (K–L) TRE calculated between the identified target points in the anatomy and the reference point in the annotation before and after alignment (K) for the example of point OLF4, and (L) for all the 26 points in main regions of interest (seeSupplementary Material S8.1for more details).

We used image subsections of the ARA_BBP_in different incidences (Fig. 6A-G;Fig. 8) to qualitatively assess whether the matching between the anatomical volume and the extended CCFv3_BBP_annotations has improved (seeSupplementary Material S8.1for more details). To evaluate the similarity between the ARA and the STPT average template before and after registration, we compared NMI scores at different stages of our pipeline: (1) before registration, (2) after rough annotation alignment, and (3) at the end of the entire alignment process (seeFig. 3A). NMI scores were calculated (A) at the whole-brain scale (Fig. 6H), as well as (B) at every subregion level of the hierarchy (Fig. 6I), and (C) for each slice in the three conventional incidences (Fig. 6J). SeeSupplementary Material S6for more details. For (B), we presented the NMI scores only for the main brain regions, namely the main olfactory bulb, cerebellum, thalamus, striatum, hippocampal region, and isocortex. We also provided an example illustrating the TRE comparison for a given point in the list of fiducial reference points of interest (Fig. 6K). Plots for the average TRE among the remaining 26 points evaluated by 5 operators and divided into 6 regions of interest are presented inFigure 6K, seeSupplementary Material S7.2for more details.

At the whole-brain scale, NMI scores between the STPT average template and the ARA_BBP_at the end of the alignment pipeline increased by 12% compared with the ARA, half of it being brought by the rough annotation alignment (Fig. 6H). For most regions, the latter alignment was bringing almost half of the similarity improvements, except for the isocortex, where this step is responsible for only a 15% increase. The isocortex showed a relatively slight TRE decrease of 72 µm (Fig. 6L). Although difficult to identify visually in the ARA_BBP_, the cortical regions alignment improved by a small margin. Each of the main brain regions presented a similarity increase between 1% and 3%, whereas the main olfactory bulb’s NMI score increased by 12% (Fig. 6I). Qualitative analysis of the labels on the ARA and ARA_BBP_inFigure 6A-Bassessed a significant improvement in the poorly aligned regions. For instance, even the innermost layer (MOB, granule layer) extended beyond the MOB, outer plexiform layer in the raw ARA (Fig. 6A). In the improved version, each distinct layer has its own texture and contrast with its adjacent layers that are consistently matching the CCFv3 annotation boundaries in the aligned version. In addition, TRE between points from that region presented one of the highest decrease of 137 µm on average (Fig. 6L), also emphasizing the improvement in the alignment. Similar improvement was measured in the cerebellum. Qualitative analysis inFigure 6F-Gshowed some substantial tissue displacement, in particular for the most lateral lobule (paraflocculus) and the most rostral lobule (lobule II). TRE reduction for this region reports the best improvement among all regions, resulting on average in a 144 µm decrease after alignment (Fig. 6L). However, some native artifacts are still present in the aligned version ARA_BBP_for that particular region, for example, the vertical distortion of the tissue on the most caudal part of the region inFigure 6G. Note that this artifact is slightly smoothed by the alignment process. Consequently, the entire cerebellum has become less distorted than the raw version. The striatum also improved its positioning in the CCFv3 annotations as it showed a significant TRE decrease of 140 µm on average (Fig. 6L). With regard to the hippocampal region, only a slight improvement was observed, before the outermost 2% increase in NMI, and an average decrease of 33 µm in TRE (Fig. 6I, L). Slight alignment improvements were measured in the dentate gyrus which is visible from the sagittal view (Fig. 6C), also reducing edge artifacts through the reduction of the distortion effect caused by the coronal tissue slicing. From a horizontal perspective, significant alignment improvements were observed for this thin region (Fig. 6D).

Focusing on areas with higher contrasts, we can assess at least a slight and at best major improvement of the matching between the ARA_BBP_and the CCFv3 annotations compared with the raw ARA in regions under review (Fig. 6A-G). Results regarding the slice-to-slice correspondence between the ARA and the STPT average template before and after alignment showed NMI increase. The similarity with the STPT average template slices on average increased by 4% in the coronal incidence and by 6% in the sagittal and horizontal incidences (Fig. 6J). Quantitative validations came to the same conclusion, both for the analysis of similarity, which increased overall in each region (Fig. 6H-J, F), and for the TRE, which decreased on average across the surveyed regions (Fig. 6L).

The anatomical data added to extend and complete the olfactory bulb and cerebellum exhibited a high degree of continuity with the surrounding regions (Fig. 6A-B, G). The extended main rostral part of the main olfactory bulb nevertheless remained blurred, making the junction between the raw and the added tissue visible (Fig. 6A-B). Continuous connection and compatibility with the caudal part of the raw ARA was achieved despite high distortion (Fig. 6G).

Results for additional alignments of the NisslSAG and the NisslHOR in the CCFv3_BBP_are presented inSupplementary Material S8.2.

### 3.2 Development of the extended CCFv3 _BBP_ annotation

The annotation brain volume changed between the different versions from the AIBS to ours: from 499.9 mm^3^for the CCFv2 to 504.9 mm^3^for the CCFv3, and finally to 511.7 mm^3^for the CCFv3_BBP_(Fig. 2A-C). The volume increased by 1.35% between the latest version from the CCFv3 and CCFv3_BBP_, with the new version being closer to literature volume for the adult mouse brain, 508.9 mm^3^(Badea et al., 2007). The new version incorporates an addition of 0.48 mm^3^in the main olfactory bulb (2.6% of its total region), 2.52 mm^3^in the cerebellum (4.5% of its total region), and 3.83 mm^3^in the medulla (11.0% of its total region), seeTable 1andFigure 7. The enlarged brain parts led to an increase of nearly 1 mm (950 µm) along the rostro-caudal axis, out of which 350 µm was dedicated to the main olfactory bulb, and 600 µm to cerebellum and medulla. In the main olfactory bulb, a larger area was affected by the extension, as part of the most rostral CCFv3_BBP_annotations was modified to incorporate its layers (Supplementary Material S9). The total mouse brain length along the rostro-caudal axis increased from 13.2 mm to 14.1 mm, becoming closer to literature length, around 15 mm (Badea et al., 2007). All the new annotations incorporated in the CCFv3_BBP_annotations benefited from qualitative and quantitative assessment using the expert annotation. In particular, high dice scores in all regions (between 0.94 and 0.99) confirmed the close correspondence between those regions in the CCFv3_BBP_annotations and the expert annotation (seeSupplementary Material S3.5).

**Table 1.** Description of the number of voxels that were added and/or modified, as well as their proportion relative to the total volume of the main olfactory bulb (MOB), the cerebellum (CB), the medulla (MY), as well as the arbor vitae (at 25 μm isotropic resolution).

	MOB	CB	MY	arbor vitae	TOTAL
Added (number of voxels)	30,870	161,026	244,784	302	436,982
Added (mm³)	0.4823	2.5160	3.8248	0.0047	6.8278
Added (% of the region)	2.6%	4.5%	11.0%	0.1%	1.3%
Modified (number of voxels)	345,440	3,291,636	592	0	3,637,668
Modified (mm³)	5.3975	51.4318	0.0093	0.000	56.8386
Modified (% of the region)	29.1%	91.1%	0.1%	0.0%	11.1%
Total (number of voxels)	376,310	3,452,662	245,376	302	4,074,650
Total (mm³)	5.8798	53.9478	3.8340	0.0047	63.6664
Total (% of the region)	31.7%	95.6%	11.1%	0.1%	12.4%

Added and modified voxels are defined as follows: (1) An added voxel is one that was unlabeled in the CCFv3 annotations but received a label in the CCFv3_BBP_annotations, (2) a modified voxel is one that was labeled in the CCFv3 annotations but changed in the CCFv3_BBP_annotations.

**Fig. 7.**
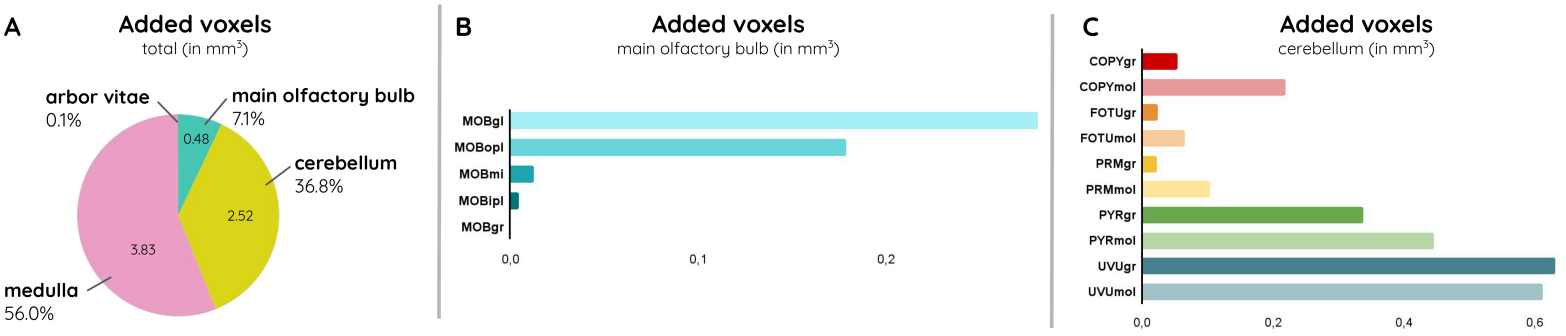
Detailed description of the added voxels (A) among all regions of the brain, (B) for layers of the main olfactory bulb, and (C) for cerebellar layers (at 25 μm isotropic resolution).

In the main olfactory bulb (Fig. 2F), it is possible to distinguish the different layers and their continuity in the entire region. Furthermore, layers in the extended part of the main olfactory bulb aligned well with the raw ARA cell distribution (Fig. 6A-B). A similar sectioned view of the cerebellum inFigure 2Gmade it possible to clearly distinguish the continuous boundaries between the granular and the molecular layers. The external layer of the extended part of the cerebellum annotation plus the boundary between the granular and molecular also fit well with the corresponding Nissl-stained areas inFigure 6F-G. The annotation boundaries were unaffected and preserved their smoothness despite the distortion artifact that remains in the caudal part of the cerebellum inFigure 6Gwhich was not fully corrected by the alignment process.

### 3.3 Generation of the average Nissl template and computation of cell distribution in the CCFv3 _BBP_

**Fig. 8.**
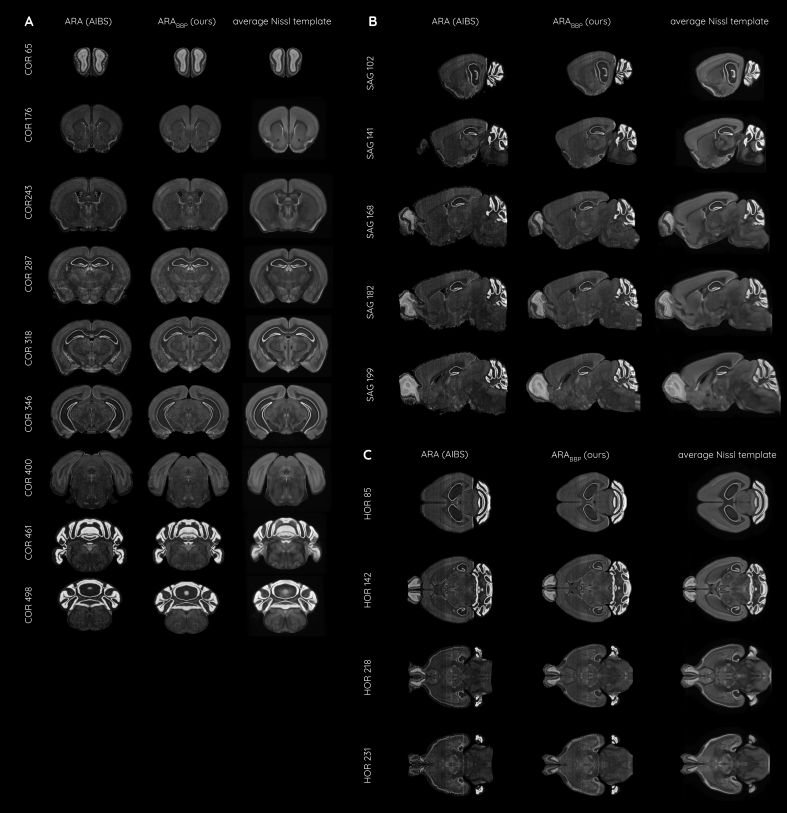
Overview of the three volumes ARA (raw), ARA_BBP_(our alignment), and the average Nissl template (including more than 86,000 registered coronal Nissl-stained slices from 734 mouse brains) in the (A) coronal, (B) sagittal, and (C) horizontal incidences.

**Fig. 9.**
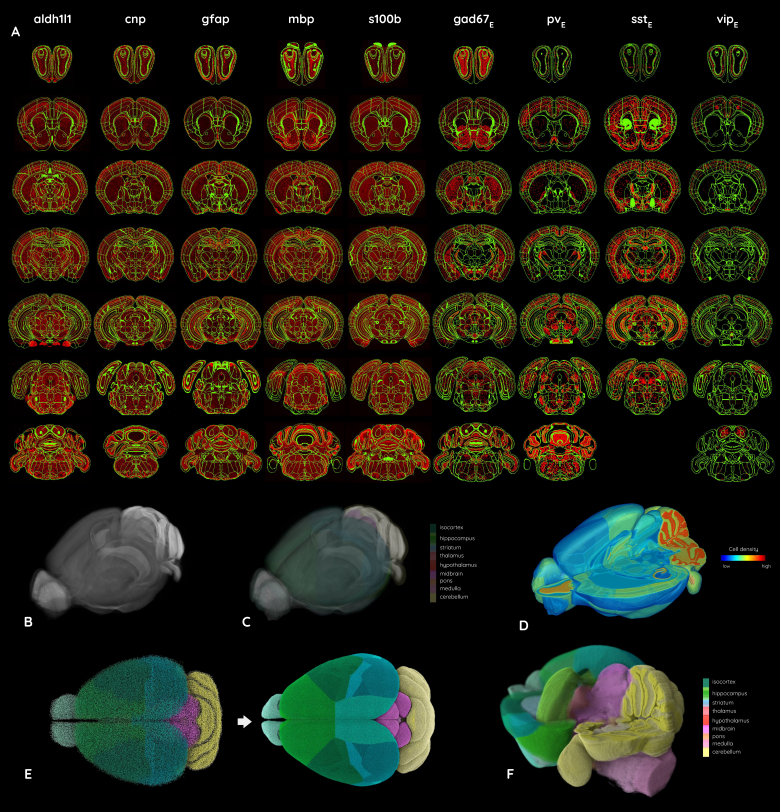
Application of the CCFv3_BBP_atlas in producing (A) the alignment of coronal slices showing gene expression for nine different genes (in red) from the ISH data portal produced by the AIBS, with the CCFv3_BBP_annotations overlaid (in green), necessary data for producing the Blue Brain Cell Atlas, (B) the average Nissl template with (C) the corresponding annotations overlayed on it, (D) the cell density per anatomical region in the entire mouse brain, (E) an enhanced neuron distribution among the brain regions in color, and (F) a 3D specific view of the neuron distribution of the entire mouse brain using colors consistent with the AIBS’s regional color code.

By combining more than 86,000 registered coronal Nissl-stained slices from 734 mouse brains, we produced an averaged Nissl-stained volume aligned in the CCFv3_BBP_from our aligned version ARA_BBP_(Figs. 6A-G,8,9A-B). The initial dataset was constructed by averaging the ARA_BBP_, NisslSAG_BBP_, and NisslHOR_BBP_(seeSupplementary Material S8.2), following the correction of significant tear artifacts in the ARA_BBP_cerebellum (seeSupplementary Material S9.2B). This averaged Nissl-stained volume exhibits enhanced smoothness (Fig. 8), effectively reducing the artifacts caused by coronal sectioning. Certain structures, such as the ventral posteromedial nucleus of the thalamus and the corpus callosum, are now more distinctly defined than in individual brains (Fig. 6D), as is the junction between the layers 4, 5, and 6 of the isocortex. Although some tissue damage remains visible, overall artifact prevalence is significantly reduced. In particular, the tissue tear in the lateral isocortex near the hippocampus was successfully corrected (Fig. 6D), and most tear artifacts in the caudal cerebellar lobules were removed (Fig. 6F-G). The precise alignment of 2D histological slices with the ARA_BBP_allowed for the visualization of its generic anatomy in 3D from a unique perspective, enabling region-specific cell labeling (Fig. 9B-C).

Most of the aligned slices fit well with the CCFv3_BBP_annotation boundaries, facilitating marker-based quantitative analyses. From these cell density distributions, we determined the coordinates, label, and type of all cells within the CCFv3_BBP_. A simulated Nissl-stained volume was produced from these data for the entire mouse brain, representing each soma with a sphere. Additionally, the extended and aligned ARA_BBP_enabled the registration of gene expression datasets from the*in situ*hybridization data portal at the AIBS (Lein et al., 2007;Ng et al., 2007). We selected a series of coronal slices, registered them to the CCFv3_BBP_atlas using the DeepAtlas suite (Krepl et al., 2021), and subsequently processed the data using the cell density estimation pipeline (Erö et al., 2018;Rodarie et al., 2022). The selected genes, which allowed for effective classification of cell subtypes, included aldehyde dehydrogenase 1 family, member L1 (aldh1l1); 2’,3’-cyclic nucleotide 3’ phosphodiesterase (cnp); glial fibrillary acidic protein (gfap); myelin basic protein (mbp); S100 protein, beta polypeptide, neural (s100b); and the expression of glutamate decarboxylase 1 (gad67E); parvalbumin (pvE); somatostatin (sstE); vasoactive intestinal polypeptide (vipE). Mapped patterns of selected gene expression aligned in the CCFv3_BBP_atlas are presented inFigure 9A.

**Fig. 10.**
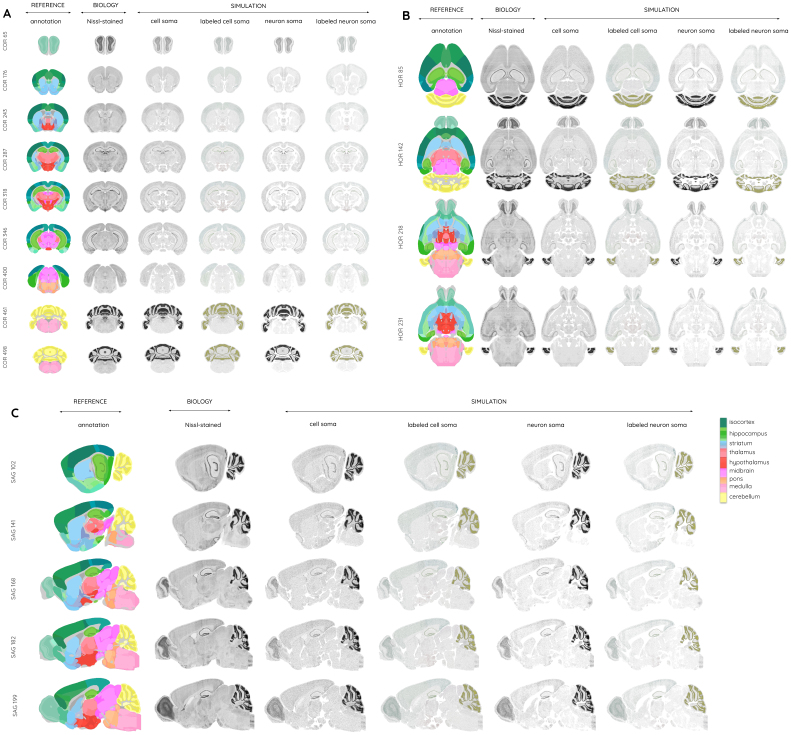
An overview of the simulated cell/neuron somas compared with real biological tissue (ARA_BBP_) from which it has been generated. These are merged with the CCFv3_BBP_annotation colors in the (A) coronal, (B) horizontal, and (C) sagittal views.

From this dataset, a simulated neuron distribution atlas was produced within the CCFv3_BBP_(Fig. 9D-F). The refined alignment of the ARA_BBP_significantly enhanced cell distribution mapping within the CCFv3_BBP_, as clearly observed inFigure 9E. The cell densities were calculated by leveraging 2D coronal slices, the new CCFv3_BBP_atlas, particularly through the improved alignment of the ARA_BBP_, providing a more accurate foundation for reconstructing and modeling cell distribution across the entire mouse brain (Fig. 10). This enabled the transformation of*post mortem*anatomical data into a framework suitable for*in silico*modeling, facilitating quantitative computational analyses and simulations. The obtained cell densities across the entire brain and within the main brain regions were validated for the principal cellular types (total cells, neurons, glia, inhibitory neurons, and excitatory neurons) by comparison with state-of-the-art reference values in the Blue Brain Atlas pipeline.

To further complete the mouse brain model, we incorporated the barrel column annotations in the isocortex into the extended CCFv3_BBP_annotations (Fig. 2D). Additionally, we connected the spinal cord annotation and its corresponding cell densities to the caudal brain, creating a fully continuous central nervous system annotation. As a result, the reconstructed mouse central nervous system atlas now spans seamlessly from the most rostral part of the main olfactory bulb to the terminal end of the spinal cord (Fig. 2D).

## 4 Discussion

We generated a new mouse brain atlas derived from the AIBS CCFv3, which includes the reconstruction and annotation of previously missing tissue in the rostral and caudal regions. Continuity and compatibility between the raw ARA and the extended tissue were assessed. Furthermore, we refined the positioning of the ARA within the CCFv3 framework, leading to a substantial improvement in its spatial correspondence with the CCFv3 annotations. Additionally, molecular and granular layer annotations were introduced into the CCFv3_BBP_, fitting the layer-specific structures in the ARA_BBP_Nissl-stained volume. These advancements facilitated the creation of a smooth, averaged Nissl-stained reference volume, as well as a simulated 3D Nissl-stained mouse brain volume, in which the position and anatomical identity of each cell can be computationally identified. Incorporation of spinal cord annotations further extended the atlas’ coverage to the central nervous system. Finally, we demonstrated two principal applications of this updated atlas: (1) the production of the first averaged Nissl template encompassing the entire mouse brain and (2) the refinement of cell distribution estimation across the whole brain.

### 4.1 Adding the missing anatomical regions in the ARA _BBP_ Nissl

To incorporate the missing anatomical regions in the Nissl-stained volume, we had to meet both targets: use biological, original Nissl-stained data and correspond to the geometry given by the CCFv3. Rigid registration, our first step, was not sufficient for creating a correspondence between the NisslSAG or the NisslHOR and the ARA_BBP_. We thus used affine registration for making the volumes correspond despite their different sectioning artifacts. An affine registration was performed at the whole-brain scale to maintain all structural correspondences between volumes, including the continuity beyond the range of the ARA_BBP_. This constraint made it possible to preserve the 3D consistency of the reconstructed anatomy in the CCFv3.

The new regions added to the ARA_BBP_volume were successfully labeled in the main olfactory bulb, cerebellum, and medulla. The main olfactory bulb labels were subdivided into five layers and the cerebellum labels were further subdivided into lobules, and then into three layers corresponding to their respective leaf regions. The medulla did not benefit from such subdivision, as the NisslHOR did not provide sufficient anatomical information to identify its leaf regions in the added tissue. To achieve this level of detail, it will be necessary to assess such subdivisions under expert supervision integrating new data including specific markers in the future.

The three Nissl-stained volumes were registered in the same CCFv3 coordinates to complement each other and to reconstruct missing tissue. These additions provide a comprehensive coverage of the entire mouse brain, each volume constructed predominantly of horizontal, sagittal, or coronal sections (seeSupplementary Material S8.2). After the construction of the extended ARA_BBP_, several regions needed to be considered carefully. Depending on the viewing incidence, the tissue appeared blurred in the extended regions. Hence, for any slice-to-slice registration, we recommend selecting the volume cut in the same incidence in which was used for producing the experimental slices. This would result in an even better alignment by ignoring the distorted 3D reconstruction effect.

As the volume dimensions increased along the rostro-caudal axis of the brain, the zero origin coordinate along this axis was shifted. Since this would change the reference space coordinate initially defined by the AIBS, we chose to include a negative shift of 350 µm in the rostral direction. This way all coordinates from the original CCFv3 were preserved.

### 4.2 Adding the granular and molecular layers in the cerebellum

The automated registration pipeline we proposed for aligning the ARA within the CCFv3 on a region-by-region basis was insufficient for accurately registering the cerebellum due to several factors. First, the absence of distinct labels differentiating the granular and molecular layers in the CCFv3 annotations complicated the alignment process. The defined hierarchical level of cerebellar lobules in the CCFv3 annotations was not precise enough to segment the ARA adequately, making accurate cerebellar region registration within the CCFv3 difficult. Second, the combination of inter-individual variability and averaging in the STPT average template resulted in a blurred anatomical separation between the granular and molecular layers, making these layers impossible to identify within the tissue. Lastly, the distorted nature of the CCFv2 annotations in this particularly folded region further added to the complexity of the registration process. Overall, a dedicated method was needed for the cerebellum, involving the identification of the granular and molecular layers before running the last monomodal registration step (seeFig. 3A).

The cerebellar cortex is composed of lobules that are subdivided into molecular and the granular layers in the CCFv2 annotations, whereas in the CCFv3 annotations, lobules are not divided. Our method introduced clear layer distinction for each lobule in the CCFv3 annotations. Using lobule annotation registration, we could have just added the granular and molecular labels from the CCFv2 annotations to the CCFv3 annotations in the cerebellum. However, as those labels were distorted, we preferred to recreate them based on Nissl-stained data, where they are clearly identifiable. This also improved the 3D smoothness of the CCFv3 annotations. In our new annotation version, the layers appeared realistic due to our data-driven method. We paid particular attention to preserving the labels of the lobules and the arbor vitae regions. This was done to maintain a shared reference coordinate system for the scientific community. We only incorporated the granular and molecular layers of the cerebellar lobules to the volume, leaving the rest unchanged.

Due to the higher cell density in the granular layer compared with the molecular layer, the boundary between these layers was clearly visible in single-brain Nissl-stained volumes. However, this characteristic became undetectable in the STPT average template, which is derived from the average of more than 1000 brains. This boundary may vary for each brain due to inter-individual variability in that region and potential artifacts that often occur on the outer edge of the tissue during extraction. Registering Nissl-stained lobules with the STPT average template lobules was difficult because the process often distorted either the granular or molecular layer to match the smooth, featureless STPT average template lobule. As a result, the lack of contrast rendered the STPT average template unsuitable for aligning the cerebellar Nissl-stained tissues. Since we were, therefore, limited to only using the annotation files (CCFv2 and CCFv3 annotations) for the lobules, we selected a more permissive and powerful registration algorithm in this region. The SyNAggro transformation (fine-scale matching and more deformation) coupled to the demons similarity metric from ANTs was chosen. The task was brought to match two different empty shapes with no contrast inside (such as in the first initialization step from the automated registration method). Since the transformation was computed for each voxel individually, the ARA anatomy also conforms to this overall deformation.

The reconstructed NisslHOR was chosen as the best dataset for automatically identifying the granular layers throughout the entire cerebellum. In particular, the NisslHOR was smoother across all three incidences and exhibited fewer artifacts in certain lobules compared with the ARA. As a result of this selection, the granular layer annotation added to the CCFv3_BBP_is free from the artifacts present in the ARA. This implies that any monomodal registration applied to our extended ARA_BBP_can be used to identify cerebellar layers in other Nissl-stained volumes.

### 4.3 Aligning the ARA in the CCFv3

Registering the CCFv2 annotations, the CCFv3 annotations, the STPT average template, and the ARA dataset in various ways presented several significant challenges. First, we attempted to register the CCFv2 to the CCFv3 annotations. A preliminary rough alignment at a comparable ontological level slightly enhanced the registration quality when applied to the ARA volume despite the significant inherent constraints posed by the distortions and discontinuities in the CCFv2 annotations. This improvement, though modest, was valuable as an initialization step because it reduced distortions in the CCFv2. Second, our efforts to register the ARA to the average template highlighted challenges when applying standard nonlinear whole-brain registration approaches to this specific multimodal alignment task. Achieving a global optimal registration was challenging due to the need to simultaneously optimize nonlinear 3D transformations across tens of millions of voxels, further complicated by variations in contrast and anatomical multimodality. The distinct modalities and dimensionalities (2D histology for the ARA versus 3D serial two-photon tomography for the STPT average template), as well as the fact the ARA is a single brain while the STPT average template is an average of 1,675 brains, further exacerbated the challenge. These difficulties motivated the development of a regionally adaptive approach, which was designed to address the anatomical and contrast-specific complexities encountered in this dataset. Third, the fundamentally different modalities of anatomical tissue versus descriptive labels interpreting regions made direct comparison and alignment unfeasible, as the ARA alone could not consistently establish clear region boundaries. Additionally, the lack of a suitable similarity metric for this hybrid registration problem highlighted the absence of adapted tools necessary for effective registration. All these challenges led us to propose a region-based registration method for registering one atlas with another.

We attempted to apply the exact same method at 10 µm isotropic resolution and assessed that similarities between the STPT average template and the ARA improved. However, some regions presented poorer registration quality than the 25 µm version. Registering regions with an even higher number of voxels made the registration algorithm struggle to estimate a global transformation that maximizes coverage between highly different contrasts within images. At this resolution, differences in contrast were heightened and their variety increased, increasing the potential for misalignment. Moreover, the 10 µm region-by-region alignment process and its validation required a much longer computing time. Ultimately, we chose a resolution of 25 µm for our registration pipeline. Nevertheless, we produced the 10 µm isotropic resolution version by upsampling the deformation field rather than computing it from the native datasets. We assumed that the low amplitude of the deformations would ensure a smooth and coherent registration process. As a result, contrasts present in the native ARA data were preserved and it was not blurred due to oversampling.

The AIBS annotation expertise made it possible to perform focused registration of the same anatomical tissue within the two volumes (region-to-region registration). This method has the advantage of preserving the alignment process of the influence of contrasts in neighboring regions, ensuring that no point outside the concerned region can be registered from the moving space to the reference space. This method also ensured that no region was disadvantaged relative to another during the registration process regardless of their contrast differences. On one hand, when a specific contrast was present in the same region across both anatomical volumes, the registration process ensured its preservation. On the other hand, when there was no particular contrast, meaning the region was homogeneous, virtually no deformation was applied and the tissue was simply transferred from one modality to the other. The result of the registered main olfactory bulb well illustrates this layer-based alignment process.

Misalignment or inaccuracies in parent-level mappings can propagate errors during registration, particularly in regions with significant label discrepancies between CCFv2 and CCFv3 annotations. To mitigate this risk, we performed the registration independently at both the leaf and parent levels, preventing the propagation of errors from higher hierarchical levels to more detailed regional structures. However, this independent approach carried the potential drawback of compromising tissue continuity when reconstructing the registered regions across hierarchical levels. This is also why a final monomodal registration step was introduced. This step aligned the reconstructed regional blocks (used as a reference) with the raw ARA volume (used as the moving image). The pipeline we designed prioritized a majority of contrasts better aligned at the leaf region level (86% of the reconstructed regions), while simultaneously preserving tissue continuity in neighboring areas. This final adjustment effectively corrected discontinuities that may have emerged from the independent leaf- and parent-level registration processes.

It was also important to mask the registered data in order to save only the most useful signal and remove the rest of the distorted tissue, making subvolumes smooth. This approximation was based on the assumption that most of the tissue was well registered, enough for providing key features in the reconstructed volume for the final registration process. Indeed, monomodal registration led to precise tissue alignment, which was not possible before. This last alignment step made it possible to retrieve 3D consistency without any tissue loss. The nonlinear registration step made transformations quite permissive, but at the same time transformations were constrained by the regions themselves. Furthermore, the independent final registration step resulted in realistic, smooth deformations of low amplitude while minimizing tissue deformation. It helped to ensure smooth transitions across subregions and correcting residual artifacts. This could explain why the NMI score increased by only 12%, the alignment resulting in many slight deformations among the whole 3D volume while preserving native anatomical consistency.

This region-based atlas alignment method can be applied to other atlases in similar contexts. By combining labels and anatomical tissues, our method addresses the multimodal registration issue, making a significant contribution to the field of multimodal atlas registration. The production of anatomical reference data aligned in the CCFv3 framework from various imaging modalities was recently initiated, particularly using light-sheet fluorescence microscopy and magnetic resonance imaging data (Perens et al., 2023). There is still work needed to integrate most reference atlases into a single, common coordinate framework, such as thePaxinos and Franklin (2019)or the Dorr (Dorr et al., 2008) atlases for instance.

All major brain regions reviewed showed a significant overall increase in the fitting of the ARA_BBP_with the CCFv3. However, it is important to acknowledge the limitations. The results were not uniform and varied from region to region. Quantitative results showed that the increase in NMI and decrease in TRE were less pronounced in the isocortex and hippocampus than in regions such as the cerebellum and main olfactory bulb. At this scale, the ARA_BBP_isocortex does not allow for the visual detection of all layers defined in it in the CCFv3_BBP_annotations, due to the lack of significant intensity differences. The alignment process only produced slight improvements in this region, due to poor contrast. This could explain the low improvement scores. Regarding the hippocampus, the most notable anatomical contrast specifically involves the dentate gyrus. Only precise parts of this very thin and dense region were misplaced in the CCFv3 annotations. The quantitative analysis does not fully capture the extent of alignment improvements in that region due to the limited number of evaluated points, which may account for the relatively minor global improvement calculated for the hippocampus. The main olfactory bulb was one of the most misaligned regions, which presented one of the highest alignment improvement scores with our version. The same situation applied to the cerebellum, where most lateral and caudal lobules were particularly misaligned. In practice, it was hard to preserve the 3D structure integrity of these regions, more specifically for the paraflocculus which stands apart from the rest of the brain. Both the main olfactory bulb and the cerebellum presented substantial differences in intensity (high contrast) and texture between their different layers. This made it possible to register each layer separately, retrieving this intensity and texture difference in the CCFv3. The final alignment stage enabled each layer to be placed in its proper position.

Similarity scores improved in all three conventional views following the alignment. It is interesting to note that the method resulted in only minor improvements in similarity scores in the coronal plane compared with the other two planes. This indicates that the alignment efforts have already been undertaken in the coronal plane (native cutting plane), compared with the sagittal and horizontal planes. In the end, our region-based alignment method could overcome the alignment difficulties between the ARA with the STPT average template, in particular in sagittal and horizontal views. Consequently, jagged-edge artifacts were smoothed out in these two planes.

A key challenge in validating our alignment was the absence of an absolute ground truth for the final positioning of the ARA within the CCFv3 space. To address this, we employed two complementary validation approaches. First, NMI was used to assess relative alignment improvements between images with differing contrast profiles. Second, TRE provided a localized accuracy estimate based on fiducial markers, representing the best available estimation for ground truth within this dataset. Observing more in depth, the NMI scores related to the main olfactory bulb similarity increased a lot more compared with the cerebellum after alignment. We could deduce that improvement was not high in the cerebellum because of the lack of contrasts in the STPT average template compared with the ARA_BBP_. Here we encountered a limitation of the similarity metric, which failed to accurately depict the intensity of similarity improvement in the cerebellar region due to multimodality. This is why we decided to evaluate the alignment of the ARA_BBP_with the CCFv3 using the TRE as an additional measure. To design the fiducial points of reference, we selected locations at the boundaries between regions. This approach was preferred over selecting points within regions because it is simpler and more effective in ensuring that they are easily identifiable in the 3D volume. Additionally, points placed within a region might not be as relevant for evaluating the alignment of the tissue within the annotation, as centers are less sensitive to the alignment accuracy than region boundaries.

### 4.4 Applications and perspectives of the CCFv3 _BBP_ new atlas version

The raw ARA volume included several artifacts, particularly affecting certain cerebellar lobules and the caudal part of the isocortex. Notably, the caudal cerebellum exhibited pronounced tear artifacts, which significantly disrupted anatomical continuity. To address this, we developed an improved version of the ARA_BBP_that incorporated automated corrections for these cerebellar artifacts, guided by cell density patterns characteristic of the concerned regions. While this version remains imperfect and involves localized modifications, it proved sufficient as a transitional reference volume with substantially improved anatomical precision compared with previous versions. This was evidenced by the alignment of tens of thousands of coronal histological slices, which achieved greater consistency in the cerebellar region when registered to the ARA_BBP_than the uncorrected ARA. Nevertheless, some residual misalignment from the raw ARA version persists in the arbor vitae, particularly within the declive (VI) lobule, where the white matter structure remains imperfectly matched to the Nissl-stained tissue.

The production of the average Nissl template in the CCFv3_BBP_increased the applicability of the new version of the atlas. The averaged Nissl-stained template became more compatible with other templates and could favor multimodal studies, in addition to being almost artifact free. Three essential elements were required to produce it: (1) the DeepSlice tool for precisely identifying each slice position in the CCFv3_BBP_, (2) the ANTs registration algorithm for producing massive nonlinear slice-to-slice registration, and (3) the reference data in the CCFv3_BBP_to bring all data into the atlas, which is the ARA_BBP_we produced. The initial construction of the average volume relied solely on the ARA_BBP_dataset. However, this approach resulted in the persistence of jagged-edge artifacts. This outcome was due to the heterogeneous nature of the ARA_BBP_, which consists of slices obtained from coronal, sagittal, and horizontal cutting planes together. We found that averaging the three most comprehensive datasets, ARA_BBP_, NisslSAG_BBP_, and NisslHOR_BBP_, provided a more robust initialization, significantly reducing the occurrence of jagged artifacts in the final average Nissl template. Combining only these three volumes proved sufficient to preserve tissue contrast, ensuring that the resulting template remained comparable with an individual brain. Notably, the complementary nature of the datasets acquired from the three sectioning planes minimized cutting-related artifacts: data from each incidence compensated for the jagged-edge effect present in the others. This integrated approach resulted in a smoother, more anatomically consistent average template initialization.

The smooth contrasts, combined with the clear highlighting of major structures and minimal visible artifacts, demonstrated that our alignment quality was sufficient for the registration algorithm to achieve accurate results. Indeed, the visible transitions between the raw ARA and the extended part disappeared. Despite some artifacts, misaligned tissues, and the fact that the entire pipeline was run at an isotropic resolution of 25 µm, we have shown that our alignment is robust enough for the ARA_BBP_to serve as a powerful tool for cross-space data transition at an isotropic resolution of 10 µm. The use of such smooth 3D data extends the capability to register sections in any plane or subvolume while preserving smooth reference data. This is particularly useful for the QuickNII tool (Puchades et al., 2019), as well as for registering 3D histology data produced by light-sheet fluorescence microscopy (Renier et al., 2016). Aligning all the sagittal Nissl-stained slices from the AIBS in our CCFv3_BBP_atlas would certainly refine this average Nissl template.

This paves the way for massive registration in the CCFv3_BBP_of gene expression databases such as the AIBS*in situ*hybridization data for instance (Lein et al., 2007;Ng et al., 2007). We especially performed the alignment of specific gene expression histological slices to produce the first*in silico*model of the cell distribution among the entire mouse brain in the CCFv3_BBP_framework. Most of the slices we used are well aligned, but misalignments were still found (seeFig. 9A), as the registration algorithm was prone to errors. Despite misalignments, the quantitative analysis of gene expression within the CCFv3_BBP_was validated through comparisons with state-of-the-art data (seeFig. 9A). By simulating the ARA_BBP_Nissl staining in the BioExplorer software ©, we could navigate through the brain, to see each cell and its corresponding region. It is, therefore, possible to generate any cross-section and imagine any experimental design for simulating Nissl staining of any region in 3D (see Video V1).

The extended CCFv3_BBP_framework facilitates the creation of a multimodal pool of anatomical data by integrating recent high-resolution research datasets, such as light-sheet fluorescence microscopy and magnetic resonance imaging (Perens et al., 2023;Voigt et al., 2019), alongside the Nissl-stained volume, the average Nissl template, and the STPT average template. Within a single reference space (CCFv3_BBP_), multiple anatomical volumes from different imaging modalities are now precisely aligned, representing a significant step toward the evolution of digital brain atlases. This multimodal integration shifts the conceptualization of brain atlases from a single-volume reference model to a more versatile, next-generation framework. In this emerging approach, the reference space is defined by a common set of anatomical annotations, while a collection of anatomical volumes from various imaging modalities is aligned to it and made available to the user.

Such a multimodal atlas system offers substantial advantages for atlas-based segmentation. When segmenting experimental data, the user would specify the imaging modality of their dataset, prompting the selection of the corresponding reference modality from the aligned anatomical pool within the CCFv3_BBP_. This approach ensures that the monomodal registration pipeline is adapted to the specific contrast and structural features of the experimental data, optimizing alignment precision and enhancing segmentation accuracy. Ultimately, this flexible, multimodal framework enables fast, reliable, and user-friendly atlas segmentation across a broad range of experimental imaging modalities.

We addressed a longstanding limitation associated with the inherently 2-dimensional nature of histological data, as most laboratories continue to rely on 2D tissue sections that often lack precise alignment in an atlas and smooth continuity when reconstructed into 3D space. The tools we developed for the automatic alignment of coronal slices within our reference atlas establish a foundation for fast and accurate atlas-based segmentation. Furthermore, the average Nissl template we produced represents a unique dataset, precisely depicting cell density and anatomical organization across the entire mouse brain within a continuous and smooth 3D volume. A key benefit of this template was the improvement in spatial accuracy of cell distribution within the Blue Brain Cell Atlas, facilitating the comprehensive reconstruction and simulation of the whole mouse brain.

The average Nissl template represents the generic cellular density across the mouse brain, enabling its simulation and the subsequent reconstruction of neuronal circuits, ultimately supporting the*in silico*modeling and simulation of the entire mouse central nervous system. The digital twin of an entire Nissl-stained mouse brain is also valuable for neuroscientists. Having captured the exact position of any experimental slice during the cutting process on the microtome made it possible to directly generate its corresponding Nissl-stained slice without having to produce it, or even directly using DeepSlice without any preliminary information. This dataset offers the same information as a dataset obtained through cell soma counterstaining.

Connecting the spinal cord to the extended annotation made it possible to propose a comprehensive CCFv3_BBP_atlas covering the central nervous system from the most rostral point of the main olfactory bulb to the end of the spinal cord. Recent advancements in tissue-clearing protocols now enable the visualization of both the mouse brain and spinal cord as a single integrated structure (Vladimirov et al., 2024). This methodological progress has driven the need for an anatomically consistent and spatially continuous reference atlas that encompasses the central nervous system. Researchers increasingly require such an atlas to facilitate quantitative analyses in the spinal cord region, while ensuring that data can be integrated seamlessly with whole-brain datasets. The incorporation of the spinal cord into the CCFv3 framework addresses this need, enabling more comprehensive central nervous system modeling and allowing for more precise investigations into neuron distribution, axonal pathways, and functional connectivity between the brain and spinal cord. Importantly, providing a unified coordinate system across the brain and spinal cord fosters comparability across studies and laboratories, reducing variability in anatomical annotations and enhancing reproducibility. The present work extends the widely used CCF, integrating the spinal cord in a manner that preserves anatomical continuity and consistency, thereby offering the community a unique and continuous reference data for multi-region studies.

By integrating complementary or more detailed annotation models, such as those for the spinal cord or barrel columns, we demonstrated that any contribution can be incorporated into a unified version of the atlas. Our goal has been to develop an atlas model aligned with the one proposed by AIBS, ensuring it is comprehensive, versatile, and openly accessible, thereby enabling others to contribute their insights and further improve it. All data are widely accessible through several platforms to connect with other models and enhance visibility within the scientific community.

**Fig. 11.**
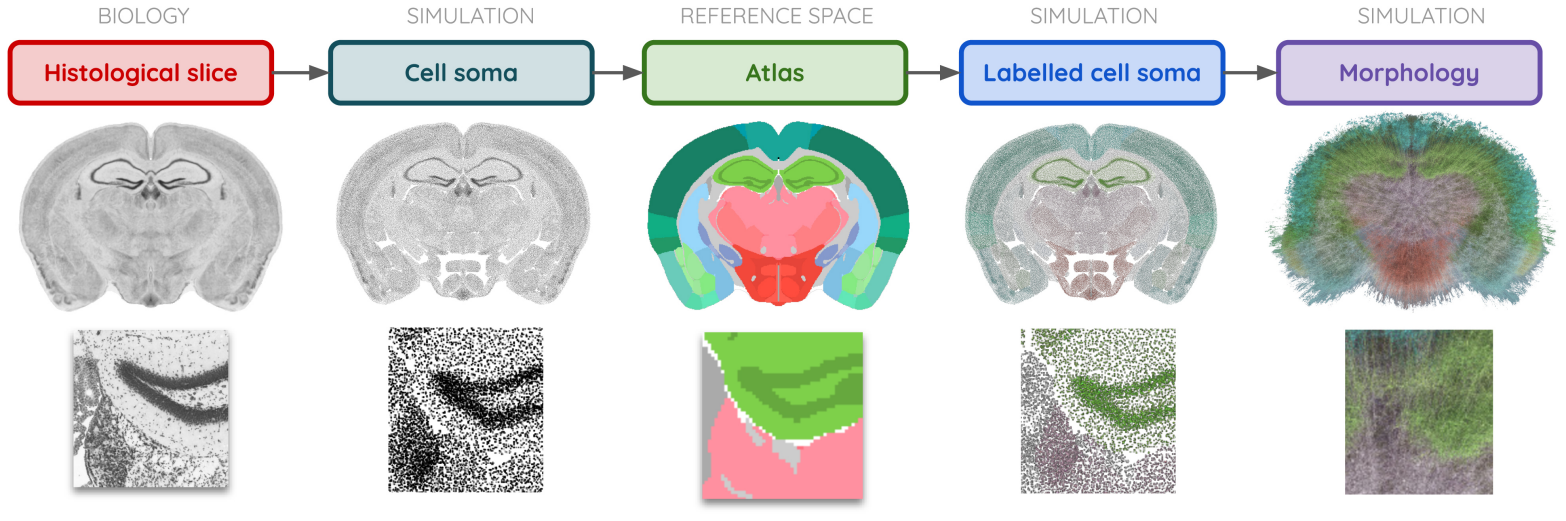
From a real biological*post mortem*coronal slice to its*in silico*model including labeled cell somas and morphologies.

The tools and data introduced in this study enable the direct transformation of real histological sections into a simulated 3D morphology (Fig. 11), with each cell assigned precise spatial coordinates, anatomical labels, and cell-type information. This cell-level anatomical framework is further connected to morphological–electrical cell types, allowing simulation of electrical activity and neuronal circuit framework within a biologically realistic model. The Blue Brain Project’s programming platform facilitates this transformation from*post mortem*biological data into*in silico*simulated brain models within a few hours, representing a major advancement in brain simulation research. Dedicated,*ad hoc*brain models can be generated from experimental histological data obtained from any laboratory, facilitating the modeling of disease-specific conditions (Fig. 10). For instance, this approach can support the simulation of neurodegenerative diseases such as Alzheimer’s disease by accurately representing neuron loss, a hallmark characteristic of its progression (Jack et al., 2010). We hope that the new atlas version and tools developed will offer other research groups a novel framework for integrating experimental data into high-fidelity computational models.

## 5 Conclusion

Our work represents a significant contribution in the field of mouse brain atlases, as we have produced an extended and improved version of the mouse brain atlas derived from AIBS CCFv3. By carefully addressing the limitations of incomplete brain coverage and alignment inaccuracies, we have reconstructed the missing tissue in both the rostral and caudal parts of the brain, while also incorporating layered differentiation in the cerebellum. Through our region-based automated and reproducible registration method, we have substantially improved the alignment of the Nissl-stained volume in CCFv3. We bridged the gaps between the native truncated CCFv3 atlas of the mouse brain and both the aligned Nissl-stained volume and the mouse central nervous system atlas. This comprehensive atlas supports a broad spectrum of applications, from histological analyses to computational modeling.

## Supplementary Material

Supplementary Material

## Data Availability

All datasets generated in this study, including the CCFv3_BBP_annotations, the ARA_BBP_, the population-averaged Nissl template, NisslSAG_BBP_, NisslHOR_BBP_, and supplementary materials, are freely available for download athttps://zenodo.org/records/15176439. A detailed description of these datasets is provided within the repository. The automated registration methods described in this study can be reproduced using the scripts available in the dedicated GitHub repository athttps://github.com/BlueBrain/ccfv3a-extended-atlas, which includes step-by-step guidelines, schematics, and implementation details. The CCFv3_BBP_atlas has been integrated into the Human Brain Project platform for use with QuickNII and VisuAlign softwares (see the zenodo repository), part of the QUINT and Locare workflows athttps://quint-workflow.readthedocs.io/en/latest/index.html, facilitating quantitative analysis and visualization of labeled features in atlas-defined regions. Additionally, the atlas is available on the BrainGlobe platform athttps://brainglobe.info/index.html, providing seamless access to computational neuroanatomy tools, including the BrainGlobe Atlas API for data processing and brainrender for high-quality 3D visualization.
