# Supplementary material for: An extended and improved CCFv3 annotation and Nissl atlas of the entire mouse brain
Source: Imaging Neurosci (Camb). 2025 May 21;3:imag_a_00565. doi: 10.1162/imag_a_00565 (PMC12319842; doi:10.1162/imag_a_00565)
Supplement: Supplementary Material [file imag_a_00565-supp.pdf]

SUPPLEMENTARY MATERIAL

S1 Detailed nomenclature, acronyms and definitions

This section presents a detailed nomenclature, defining all acronyms used throughout this supplementary material file and the associated data available on the open portal. It is intended to provide clear guidance for navigating the datasets and understanding the different versions offered, ensuring their appropriate and effective use. This detailed nomenclature is consistent with the data freely available for open-access download at the following repository: https://zenodo.org/records/14034334.

AIBS: Allen Institute for Brain Science

AIBS Nissl$_{SAG}$: whole mouse brain from the AIBS sectioned in the sagittal incidence

AIBS$_{BBP}$ Nissl$_{SAG}$: AIBS sagittal Nissl-stained volume aligned in the CCFv3a$_{BBP}$ at BBP

ANNOTv2: CCFv2 annotation volume

ANNOTv3: CCFv3 annotation volume

ANNOTv3a$_{BBP}$: extended CCFv3 annotation produced at BBP covering the entire mouse brain and including new cerebellar layers

ANNOTv3c$_{BBP}$: extended CCFv3a$_{BBP}$ annotation produced at BBP covering the mouse central nervous system including spinal cord as well as barrel columns

ANTS: Advanced Normalisation Tools

ARA Nissl$_{COR}$: Allen Reference Atlas coronal Nissl-stained volume

ARA$_{BBP}$: simplified acronym used in the manuscript for describing the BBP produced data, which represents the aligned ARA$_{BBP}$ Nissl$_{COR}$ and the extended and aligned ARAva$_{BBP}$ Nissl$_{COR}$

ARA$_{BBP}$ Nissl$_{COR}$: ARA Nissl$_{COR}$ accuratelay aligned in the CCFv3 at BBP

ARAva$_{BBP}$ Nissl$_{COR}$: ARA Nissl$_{COR}$ accuratelay aligned and extended in the CCFv3 at BBP

ARM$_A$: automated registration method for aligning the ARA Nissl$_{COR}$ into the CCFv3

ARM$_B$: automated registration method for extending the main olfactory bulb Nissl

ARM$_C$: automated registration method for extending the cerebellum as well as the medulla Nissl, plus adding the missing cerebellar granular and molecular layers.

BBP: Blue Brain Project

CCF: Common Coordinates Framework

CCFv2: Common Coordinates Framework version 2

CCFv3: Common Coordinates Framework version 3

CCFv3$_{BBP}$: Simplified acronym used in the manuscript for describing the entire mouse brain atlas and central nervous system derived from CCFv3 produced at BBP, which are CCFv3a$_{BBP}$ and CCFv3c$_{BBP}$

CCFv3a$_{BBP}$: Entire mouse brain atlas derived from CCFv3 produced at BBP

$CCFv3c_{BBP}$: Mouse central nervous system atlas derived from CCFv3 produced at BBP including spinal cord and barrel columns

NiftyRegF3D: non-linear 3D Nifty registration

NMI: normalized mutual information

STPT: serial two photon tomography

SyN: symmetric image normalization

TRE: target registration error

WAXH $Nissl_{HOR}$: whole mouse brain from the Waxholm space sectioned in the horizontal incidence

$WAXH_{BBP}$ $Nissl_{HOR}$: Waxholm horizontal Nissl-stained volume aligned in the $CCFv3a_{BBP}$ at BBP

TABLE S1.1 | Acronyms, definition, and origin of the data used and produced in this study

**AIBS**	**Allen Institute for Brain Science**
ARA $Nissl_{COR}$	Allen Reference Atlas coronal Nissl-stained volume pre-aligned in the CCFv3 by the AIBS
ANNOTv2	Annotations incorporating region delineations defined on the ARA $Nissl_{COR}$
STPT average template	Serial Two-Photon Tomography average template
ANNOTv3	Annotations incorporating region delineations defined on the STPT average template
CCFv2 = ARA $Nissl_{COR}$ + ANNOTv2	Common Coordinate Framework version 2
CCFv3 = STPT average template + ANNOTv3	Common Coordinate Framework version 3
AIBS $Nissl_{SAG}$	AIBS sagittal Nissl-stained volume
WAXH $Nissl_{HOR}$	Waxholm horizontal Nissl-stained volume
**BBP**	**Blue Brain Project**
ARA_{BBP} $Nissl_{COR}$	ARA $Nissl_{COR}$ accuratelay aligned in the CCFv3 by the BBP
$ARAv3a_{BBP}$ $Nissl_{COR}$	ARA $Nissl_{COR}$ accuratelay aligned and extended in the CCFv3 by the BBP
$ANNOTv3a_{m}$	Expert manual delineation produced by the BBP of the extended tissue based on $ARAv3a_{BBP}$ $Nissl_{COR}$ as well as of the granular and molecular layers in the cerebellum
$ANNOTv3a_{BBP}$	Extended CCFv3 annotation produced by the BBP covering the entire mouse brain plus including new cerebellar layers, assessed using $ANNOTv3a_{m}$
$CCFv3a_{BBP}$ = ARA_{BBP} $Nissl_{COR}$ + $ANNOTv3a_{BBP}$	Entire mouse brain atlas derived from CCFv3 produced by the BBP
$ANNOTv3c_{BBP}$	Extended $CCFv3a_{BBP}$ annotation produced by the BBP covering the mouse central nervous system including spinal cord as well as barrel columns
$CCFv3c_{BBP}$ = ARA_{BBP} $Nissl_{COR}$ + $ANNOTv3c_{BBP}$	Mouse central nervous system atlas derived from CCFv3 produced by the BBP including spinal cord and barrel columns
average Nissl template	An averaged Nissl-stained template in the $CCFv3a_{BBP}$
$AIBS_{BBP}$ $Nissl_{SAG}$	AIBS sagittal Nissl-stained volume aligned in the $CCFv3a_{BBP}$ by the BBP
$WAXH_{BBP}$ $Nissl_{HOR}$	Waxholm horizontal Nissl-stained volume aligned in the $CCFv3a_{BBP}$ by the BBP

S2 Data location

All data are in open access and were downloaded from the following links:
© 2015 Allen Institute for Brain Science. Allen Brain Atlas API. Available from: brain-map.org/api/index.html

- Allen Mouse brain atlas (2011):
  - CCFv2 annotation (ANNOTv2): https://download.alleninstitute.org/informatics-archive/current-release/mouse_ccf/annotation/mouse_2011/)
  - coronal Nissl-stained volume (ARA $Nissl_{COR}$): https://download.alleninstitute.org/informatics-archive/current-release/mouse_ccf/ara_nissl/)
- Allen Mouse brain atlas (2022):
  - CCFv3 annotation 2022 (ANNOTv3): https://download.alleninstitute.org/informatics-archive/current-release/mouse_ccf/annotation/ccf_2022/
  - average template: https://download.alleninstitute.org/informatics-archive/current-release/mouse_ccf/average_template/
- hierarchy file: http://api.brain-map.org/api/v2/structure_graph_download/1.json
- sagittal Nissl-stained mouse brain volume (AIBS $Nissl_{SAG}$): https://mouse.brain-map.org/experiment/show/100042147
- horizontal Nissl-stained mouse brain from the Waxholm space (WAXH $Nissl_{HOR}$): https://www.nitrc.org/projects/incfwhsmouse

S3 Pre- and post-processing on the CCFv3 latest version

S3.1 CCFv3 atlas downsampling

To create a 25 µm isotropic resolution version of the annotation volume, we used slicing to downsample the Allen Institute's 10 µm isotropic resolution volume. A set of procedures were applied to the 10 µm isotropic resolution.

First, we defined the scaling factor as 2.5 to reflect adjusting from 25 µm to 10 µm, which corresponds to reducing each dimension of the array by a factor of 2.5.

Next, we computed the new shape of the array as a tuple of integers, where each dimension is divided by the scaling factor and rounded down to the nearest integer.

Then, we used the `numpy.linspace()` function to generate a set of evenly spaced indices along each dimension of the array. The `linspace()` function takes three arguments: the start value (0), the stop value (dim-1), and the number of indices (new_dim). The resulting indices are floating-point numbers, so we convert them to integers using the `astype()` method.

Finally, we used the `numpy.ix_()` function to select a subarray of the original array using the indices. The `ix_()` function takes one or more indexing arrays as arguments and returns a tuple of arrays that can be used to index into the original array. We then stored the selected subarray in the new_atlas variable and print the shapes of the original and new arrays to verify that the new array has the desired shape of (528, 320, 456).

This downsampling step inherently introduces artifacts, as it either enlarges the relative size of brain regions smaller than a 25 μm^3 edge voxel or completely removes them from the annotation volume. If we look at the symmetric difference between the two annotation versions after slicing, only the regions field CA1 (382), field CA3 (463), retrosplenial area, dorsal part, layer 4 (545), and direct tectospinal pathway (1051) disappeared from the downsampled annotation volume. Note, that the original size (10 µm isotropic resolution) of these regions were only 6, 2, 2, and 4 voxels, respectively. While taking note, we deemed these changes acceptable, as the alternative would have entailed significantly enlarging the relative size of these small regions. Code is available upon request.

S3.2 Annotation corrections

In the original 10 µm isotropic resolution annotation volume from the Allen Institute, four isolated and discontinuous voxels, located at the extreme borders of the volume (most rostral coronal plane), were detected. We assumed these voxels were errors and removed them from the annotation file before downsampling. They were labeled as being field CA3, stratum lacunosum-moleculare (471). The coordinates of those four voxels are [1319, 799, 520], [1319, 799, 521], [1319, 799, 618], and [1319, 799, 619].

The latest version of the CCFv3 annotation is not symmetrical. In particular, the dorsal tegmental decussation (1060) contains 430 more voxels in the left hemisphere compared to the right hemisphere, all located in the most medial left sagittal plane.

Moreover, three more discontinuous points are only present in the left hemisphere, two from the main olfactory bulb, mitral layer (236) located at positions [245, 500, 524] and [245, 501, 524], and one from the Lateral septal nucleus, rostral (rostroventral) part (258) at position [548, 313, 521]. In this work, we focused only on the right hemisphere, eliminating all these points.

Part of the main olfactory bulb (507) label was re-labeled by an expert into the main olfactory bulb, glomerular layer (212) as this missing layer was part of its parent region. As the glomerular layer is the outermost layer of the main olfactory bulb, only voxels meeting this description were re-labeled.

S3.3 Manual annotation of newly reconstructed regions by experts

Manual expert annotation was performed using ITK-snap segmentation tool to complete the missing layers in the main olfactory bulb, specifically in the granular, inner plexiform, mitral, outer plexiform, and glomerular layers. Additionally, new labels were created for the granular and molecular layers across all 16 cerebellar lobules including the extended regions (see **Figure S9.1**). We based our delineation in the $ARAva_{BBP}$ $Nissl_{COR}$ on the distinct laminar structure of these regions. First, the missing annotated volume was completed with the outermost label from the ANNOTv3a, using a single view (coronal, sagittal, or horizontal) based on the presence of tissue in the Nissl volume. Second, the annotation of each layer was added in the same incidence as in the first step, based on the visual Nissl contrasts and/or textures. Third, the ANNOTv3a was corrected in each incidence to minimize discontinuities resulting in an accurate 3D annotated volume. Fourth, the ANNOTv3a was edited using 3D meshes (previews) for each layer to ensure there were no holes, resulting in a smooth volume. The goal of the third and fourth steps was to achieve a good compromise for each layer between maintaining consistency with the preexisting atlas, the aligned $ARAva_{BBP}$ $Nissl_{COR}$, and the new manual labels produced.

S3.4 Semi-automated method for producing the labels on the newly reconstructed regions

After having produced the $AIBS_{BBP}$ $Nissl_{SAG}$ and $WAXH_{BBP}$ $Nissl_{HOR}$ aligned to the $ARAva_{BBP}$ $Nissl_{COR}$ (see **Supplementary Material S8.2**), we averaged those three volumes to get better smooth contrasts between regions. This allowed us to design a semi-automated method composed of image processing techniques as well as minimal manual corrections (**Figure S4.4**) for identifying the labels in the extended part, as well as the granular and molecular layers in the cerebellum. A dedicated method was used for each region under consideration. Concerning the medulla, a simple process was set up as only a binary identification of the tissue was performed. This region underwent extensive manual processing, including smoothing and correction, to address the substantial noise present in the WAXH $Nissl_{HOR}$ medulla. These adjustments were necessary also to ensure consistency and continuity with ANNOTv3 from the AIBS. No additional expert annotation was produced for that region. For the main olfactory bulb, a progressive growing region process was

designed, starting with the identification of the innermost region (granular layer, MOBgr) using Otsu thresholding, and ending with the outermost region (glomerular layer, MOBgl). The inner plexiform (MOBipl) as well as the mitral (MOBmi) layers were automatically estimated using a dilation with a 3 isotropic voxel kernel, in accordance with the ANNOTv3. Otsu thresholding made it possible to distinguish the outer plexiform layer (MOBopl) from the glomerular layer in the remaining unannotated tissue. After some smoothing operations, minimal manual corrections were applied for ensuring continuity between the new labels in the extended part and the ANNOTv3 for the main olfactory bulb layers. The method used for the cerebellum was different because it required identifying layers from scratch within each of its 16 lobules. We processed each of the lobules independently with the same method to provide a better precision in layer identification. Manual corrections were applied for ensuring continuity of the layers between lobules according to the ANNOTv3. Despite aligning and averaging 3 different brains, many artifacts of tearing, folding or missing tissue are still present in the data, especially in the cerebellum, which is why we had to perform manual artifact correction. Each of these corrections was performed using the Paxinos and Franklin paper atlas Fifth edition, as well as the expert delineations of the AIBS Nissl_{SAG} on the web interface from the Allen Institute as a support.

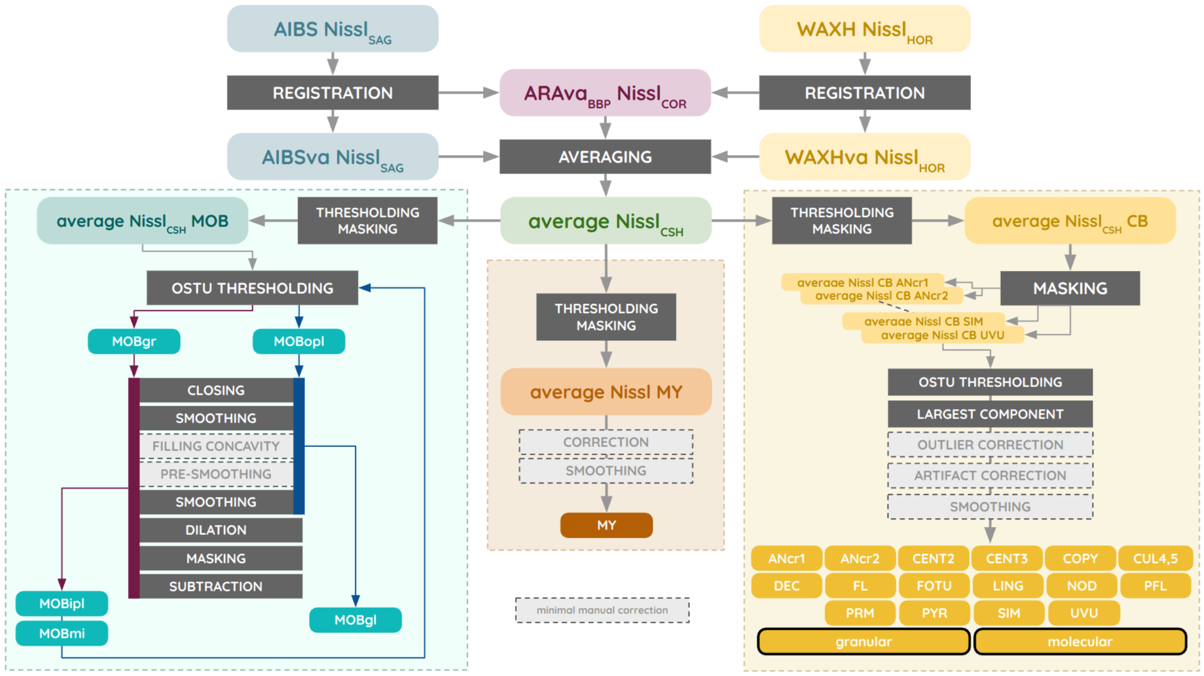

FIGURE S3.4 | Semi-automated method for identifying the labels in the extended parts of the main olfactory bulb (MOB), cerebellum (CB), and medulla (MY), as well as the granular and molecular layers in each of the 16 lobules in the cerebellum. This method is applied on the average Nissl_{CSH} derived from the three Nissl-stained volumes AIBS Nissl_{SAG}, WAXH Nissl_{HOR} and ARAva_{BBP} Nissl_{COR}.

S3.5 Assessment of the newly added labels with manual expert annotation

Visual assessment as well as Dice score calculation were performed on each of the extended/added layers in the main olfactory bulb and the cerebellum. The **Figure S3.5A** shows that the difference between the two versions, measured at the boundary between

layers, rarely exceeds a thickness of 3 voxels. Most of the differences are between 0 and 1 voxel thickness. This tends to be confirmed by the Dice score for all regions in the **Figure S3.5B**, which is 0.97 on average for the olfactory bulb and the cerebellum, the lowest score being 0.94 obtained for the mitral layer of the main olfactory bulb. All the labels generated by the semi-automated method described in section S3.4 were validated by these high scores, indicating that our method accurately reproduced the expert labels on the anatomical data $\text{ARAva}_{\text{BBP}}$ $\text{Nissl}_{\text{COR}}$ with high precision.

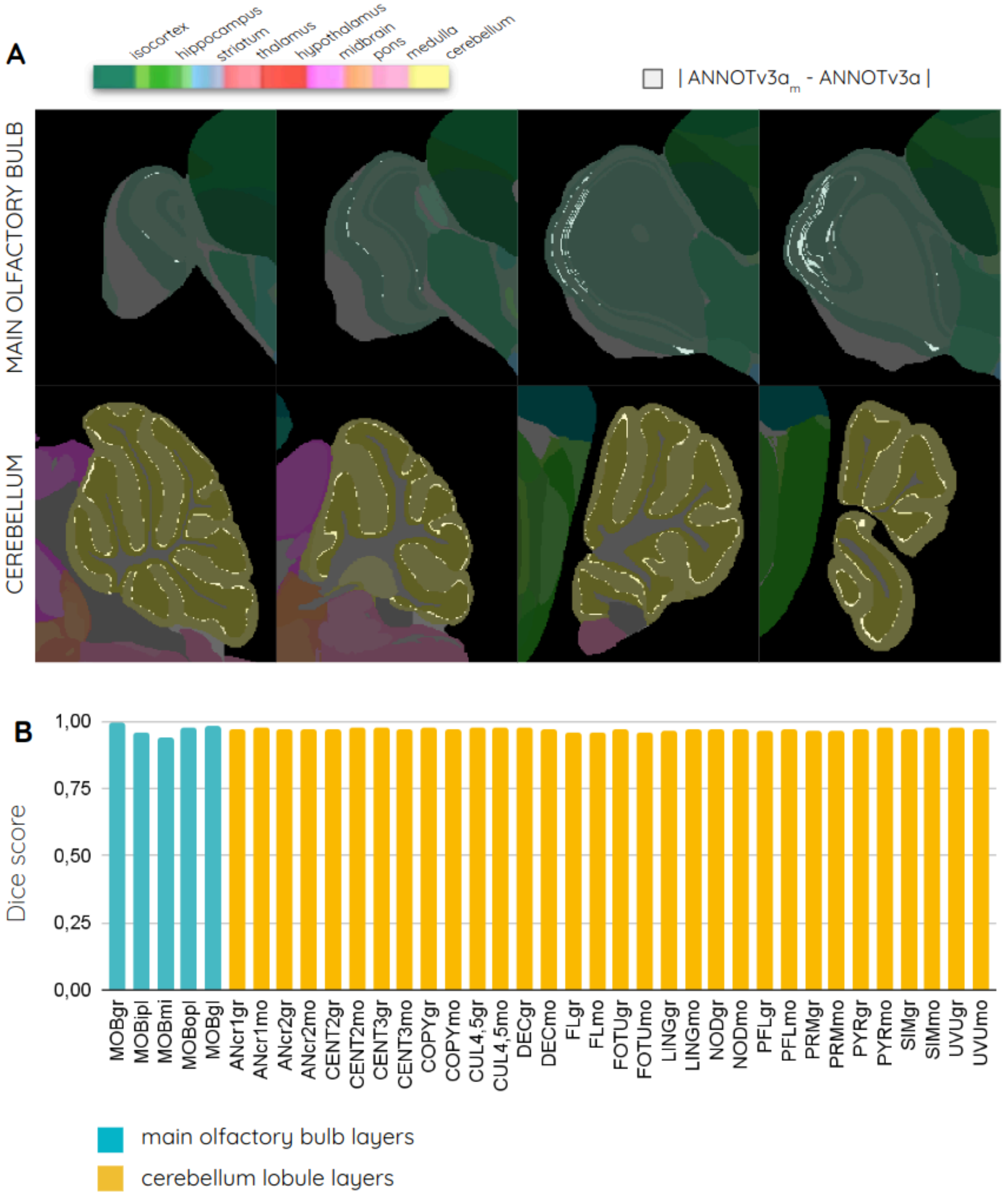

FIGURE S3.5 | Comparison between the expert manual annotation (ANNOTv3a_m) and the annotation obtained by the semi-automated method presented in section S3.4 (ANNOTv3). (A) Absolute difference between the two compared labels, and (B) Dice scores evaluated for all the extended/added regions.

S4 Detailed description of the automated registration pipeline for aligning and extending the ARA in the CCFv3

This section provides a detailed schematic overview of the automated registration methods (ARM) employed to align the ARA within the CCFv3 framework and to extend its coverage to the entire mouse brain. The full set of automated registration procedures described here is available for reproduction via the dedicated GitHub repository: https://github.com/BlueBrain/ccfv3a-extended-atlas. This repository corresponds to the nomenclature presented in **Supplementary Material S**1.

An initial schematic (**Figure S4.1**) illustrates the overall automated registration pipeline, which consists of three distinct registration methods: ARM_A dedicated to the alignment of the ARA in the CCFv3, ARM_B dedicated to the extension to the main olfactory bulb, and ARM_c dedicated to the extension of the cerebellum and medulla. Detailed schematics and descriptions of each registration method (ARM_A, ARM_B, and ARM_C) are provided in **Figure S4.2**,**Figure S4.3**, and **Figure S4.4**, respectively.

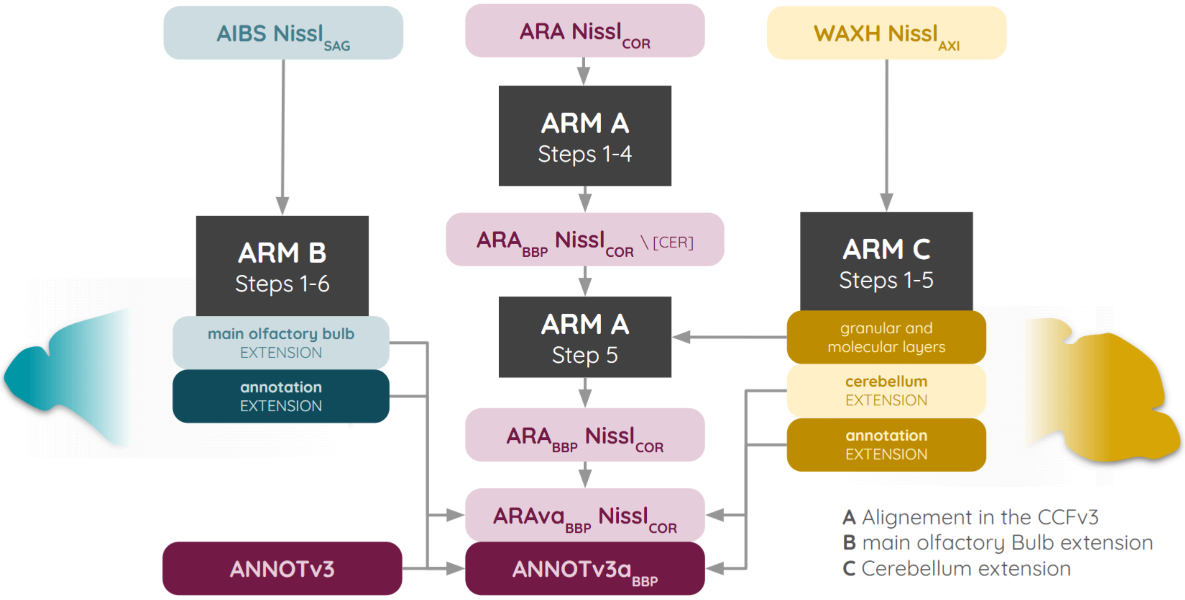

FIGURE S4.1 | Automated registration pipeline for the precise alignment of the Nissl in the CCFv3 and the extension of the CCFv3, composed of the automated registration methods ARM_A, ARM_B, and ARM_C.

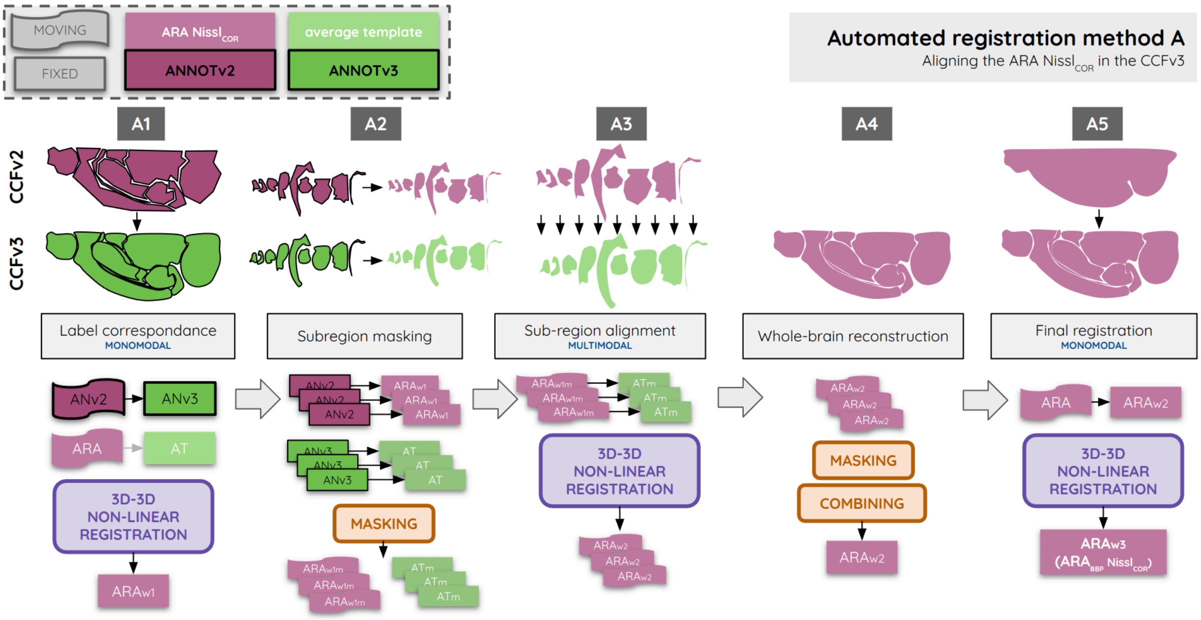

FIGURE 4.2 | The five-steps region-based guided registration method ARM_A for aligning the ARA $Nissl_{COR}$ in the CCFv3. The five steps for the ARM_A method are ranging from A1 to A5.

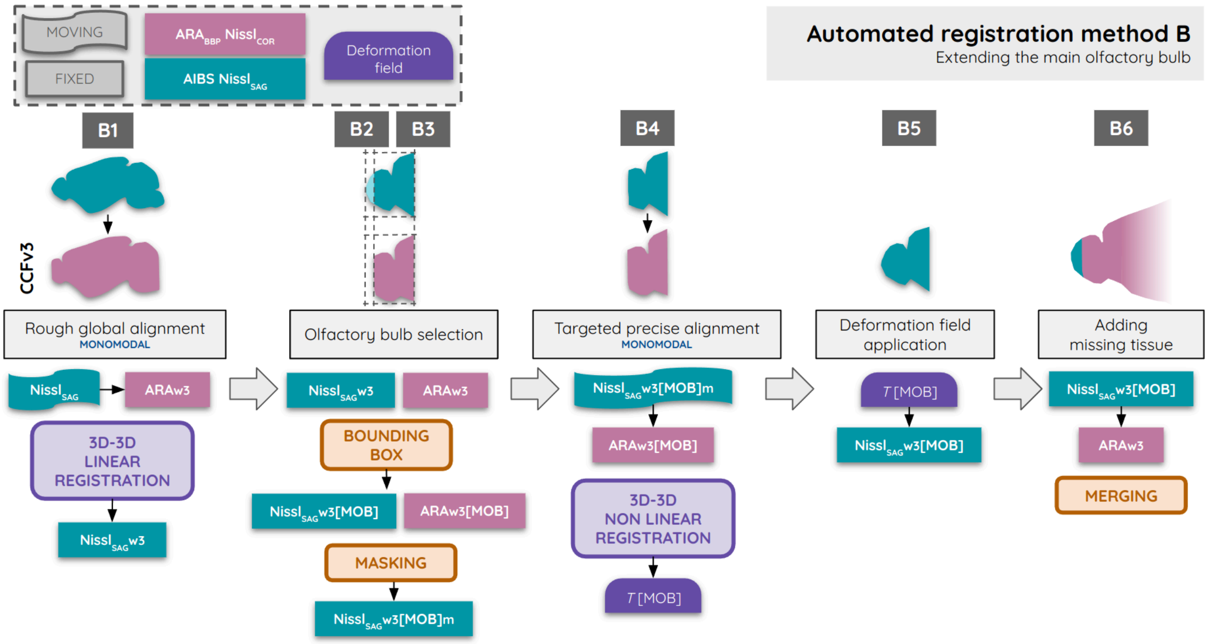

FIGURE 4.3 | The six-step automated pipeline for adding the missing tissue from the AIBS $Nissl_{SAG}$ to the ARA_{BBP} $Nissl_{COR}$ in the CCFv3 olfactory bulb. The six steps for the ARM_B method are ranging from B1 to B6.

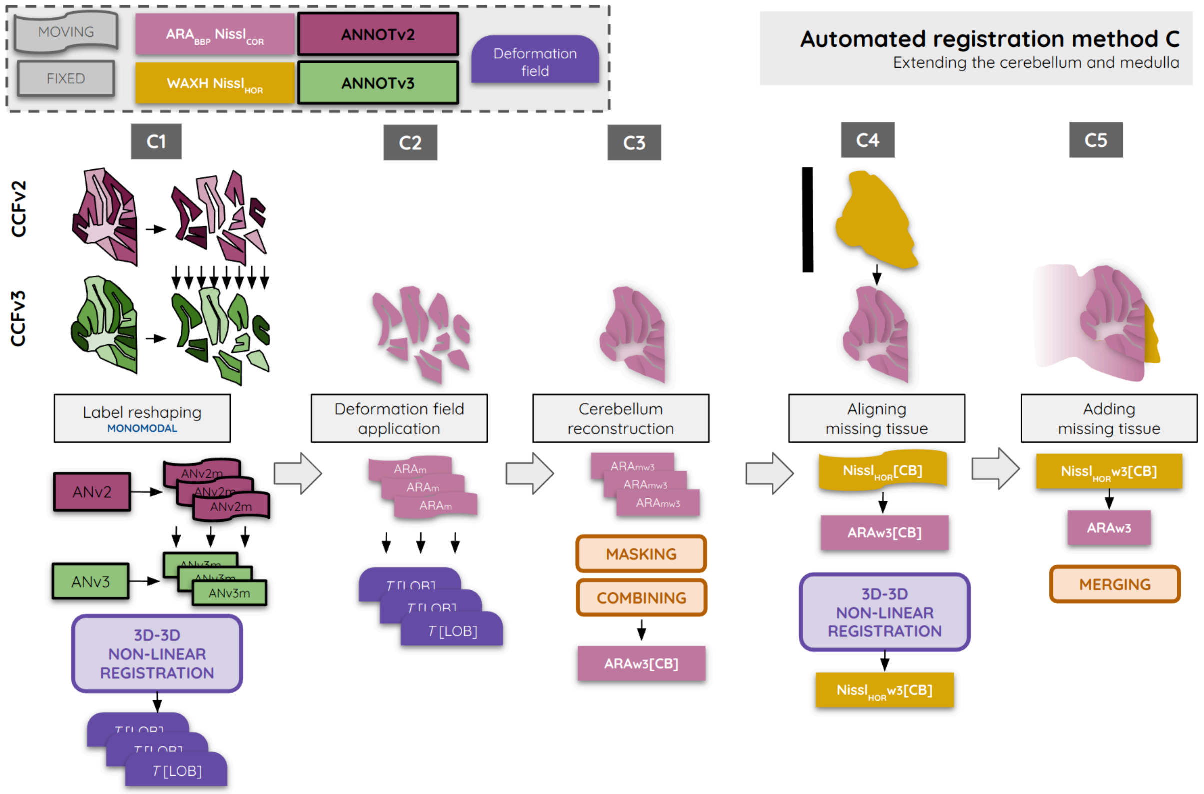

FIGURE 4.4 | The five-steps automated pipeline for adding the missing layers in the ANNOTv3 as well as the missing tissue from the WAXH Nissl$_{HOR}$ to the ARA$_{BBP}$ Nissl$_{COR}$ in the CCFv3 cerebellum. The five steps for the ARM$_c$ method are ranging from C1 to C5.

S5 Hierarchy comparison between CCFv2 and CCFv3

The two latest versions of the annotation atlas provided by the Allen Institute CCFv2 (ANNOTv2) and CCFv3 (ANNOTv3) have different numbers of leaf regions (with non-zero voxel support), while they are based on the same hierarchy file. The ANNOTv3 shows a 14% reduction in the number of existing grey matter regions, while a 7% increase in the number of existing white matter regions, compared to ANNOTv2 (**Figure S5.1A-B**). Only 527 regions are therefore comparable when we consider the existing common regions in the two versions (**Figure S5.1C**).

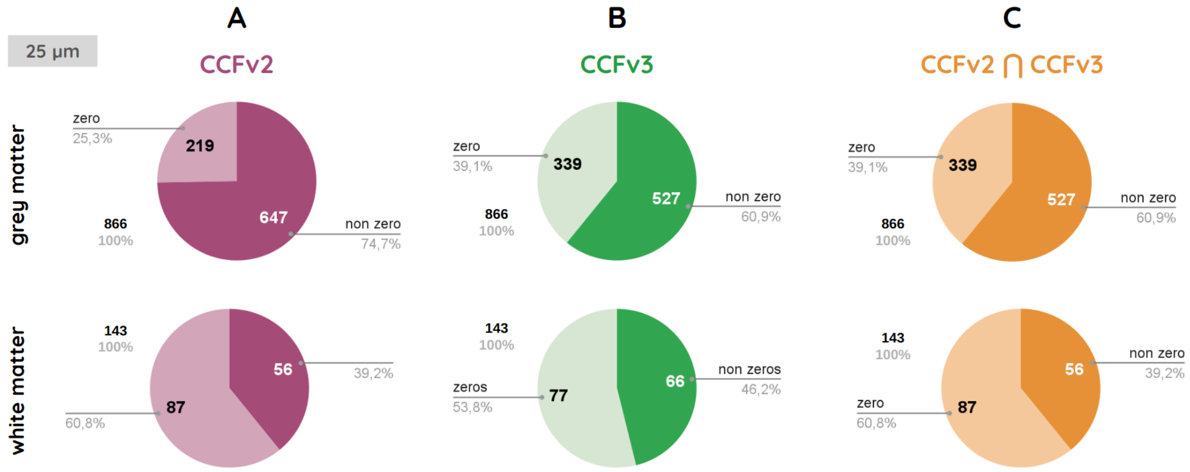

Figure S5.1 | Existing regions (non-zero voxel support) in each annotation file. (A) CCFv2 and (B) CCFv3, as well as (C) their intersection, *i.e.* the common regions in the two versions.

Since not all regions have a direct counterpart between ANNOTv2 and ANNOTv3, we defined an intermediate level of ontology (referred to as 'parent'), outlined in **Figure S5.2**.

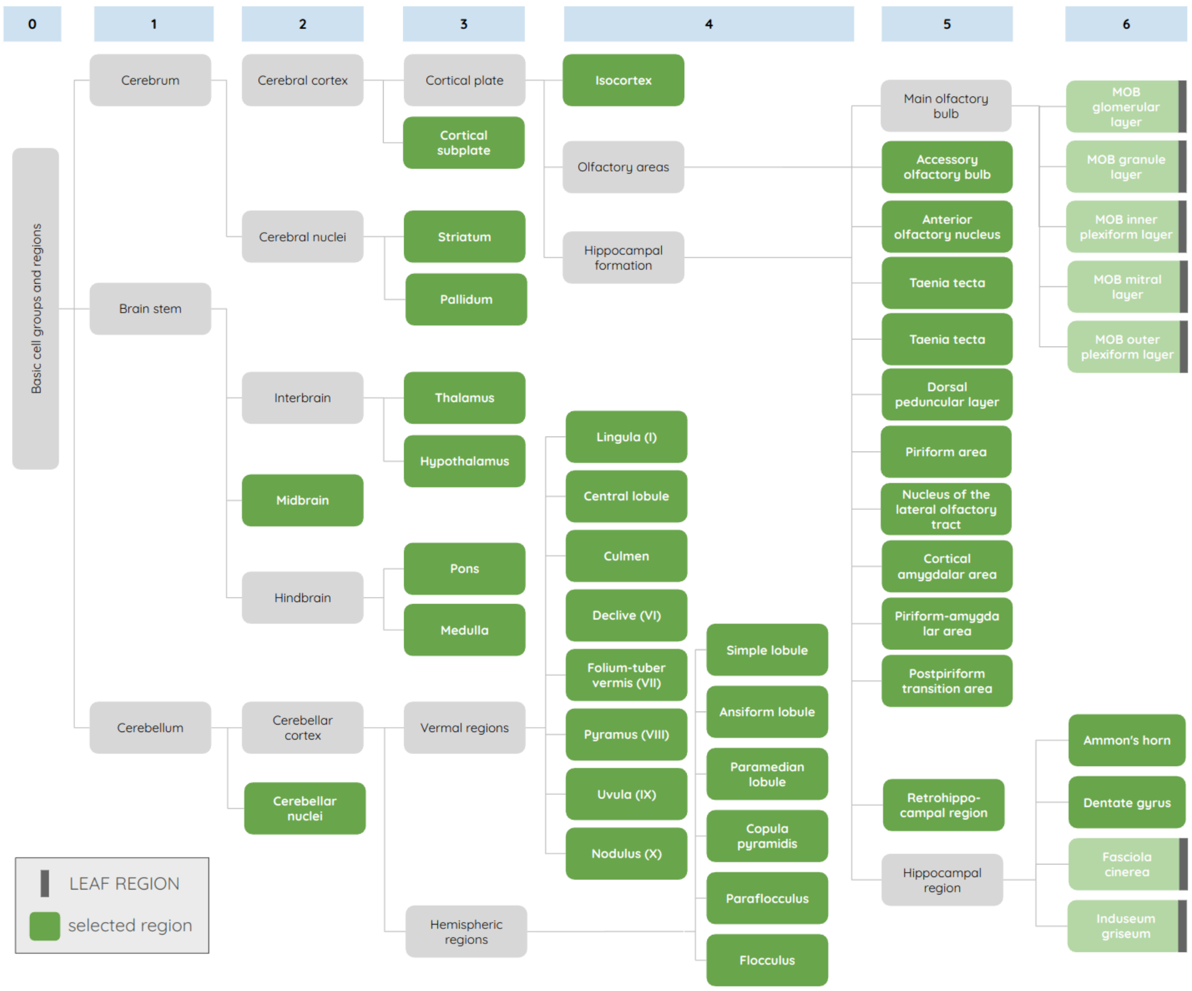

FIGURE S5.2 | Overview of the second ontology level (parent) according to the different groups of regions in the hierarchy file for the region-based registration pipeline.

S6 Slice-to-slice similarity

We provide detailed slices-to-slice Normalized Mutual Information (NMI) scores to evaluate the similarity between the template and the aligned $Nissl_{COR}$ in the three conventional incidences: coronal (**Figure S6.1A**), sagittal for the right hemisphere (**Figure S6.1B**), and horizontal (**Figure S6.1C**), comparing results before (ARA $Nissl_{COR}$) and after (ARA_{BBP} $Nissl_{COR}$ after Step A5) alignment in the CCFv3.

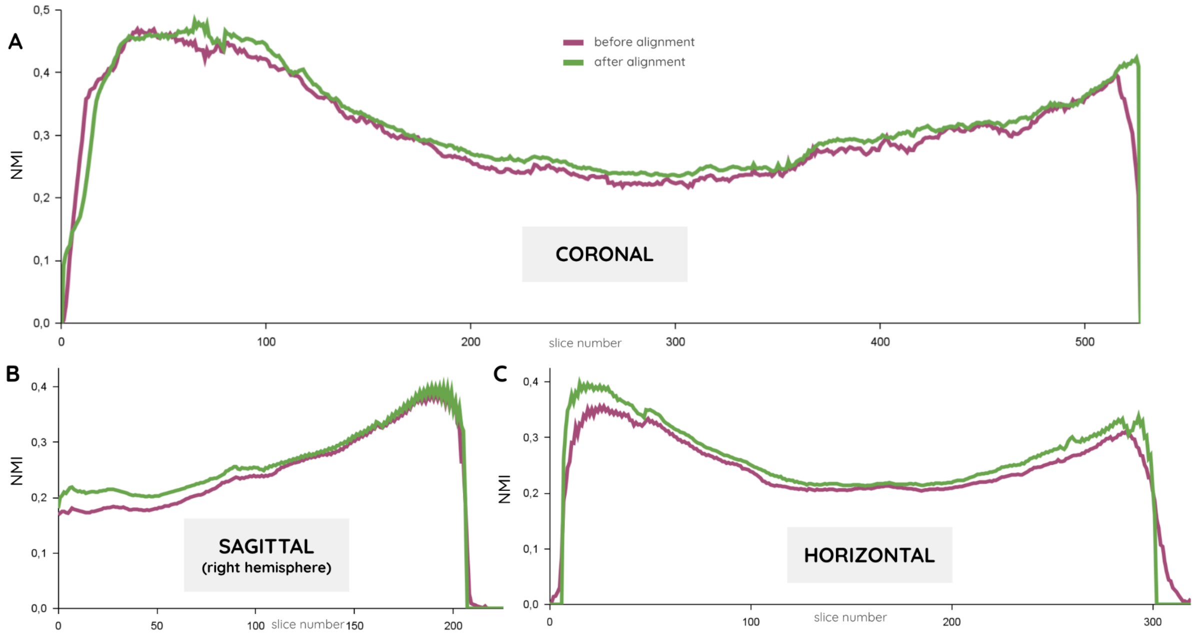

FIGURE S6.1 | Slice-to-slice normalized mutual information estimated between each slice of the template and the ARA $Nissl_{COR}$ before (green) and ARA_{BBP} $Nissl_{COR}$ after alignment (burgundy) in (A) the coronal incidence, (B) the sagittal incidence for the right hemisphere, and (C) the horizontal incidence.

S7 Point identification in the ARA Nissl$_{COR}$

S7.1 Detailed method

We provide here the list of 29 fiducial points for assessing the quality of the alignment of the ARA Nissl$_{COR}$ in the CCFv3 (**Table S7.1** and **Figure S7.2**). All points were selected for their strategic placement at the boundaries between regions, clear 3D identifiability, and broad coverage of the main regions in the brain: olfactory areas, cerebellum, hippocampus, striatum, brainstem, thalamus, and cortex. We excluded points at the external border of the brain where it meets the background. Comprehensive documentation was provided for each point, enabling any operator to identify them without requiring specialized knowledge in neuroanatomy (**Table S7.1**). This documentation includes (1) the point's coordinates in the CCFv3 right hemisphere, (2) the region name and ID in the hierarchy it belongs to, (3) the preferred viewing plane for optimal identification, and (4) a detailed description of how the point was identified in 3D, with specific reference to its relative position along the rostro-caudal, dorso-ventral and medio-lateral axis. No leaf region in the hierarchy appears more than twice. Note that when multiple candidates appeared correct, the operator was asked to always select the one located at the center of the region's surface from that perspective.

Five different operators with various backgrounds and profiles (scientists or engineers, in biology, computational neuroscience, machine learning) identified a set of 29 fiducial points in each ARA Nissl$_{COR}$ volume before (ARA Nissl$_{COR}$) and after (ARA$_{BBP}$ Nissl$_{COR}$; after Step A5) alignment in the CCFv3. Operators were asked to give a score from 1 to 5 for each point to assess how straightforward it was to identify it in the Nissl tissue, defined as being: (1) very hard, (2) hard, (3) easy, (4) obvious, or (5) quite obvious. This confidence rate enabled us to remove the points for which this score was significantly low on average across all operators (< 2), meaning that the location of these specific points was not representative of their real anatomical location according to operators' evaluation. This way, we only kept points which were easy to recognize, eliminating any potential confusion in the identification process. Additionally, we removed points presenting inconsistent distances estimated to be greater than 1 millimeter, which indicates erroneous point identification, as no such high-intensity deformation was observed in the deformation field estimated at Step A5.

TABLE S7.1 | Detailed description of the 29 fiducial points in the ANNOTv3.

Acro nym	Coordinates	Region name	ID	Incidence	Easy-to-understand description
OLF1	[55, 164, 29]	Main olfactory bulb, inner plexiform layer	228	HORIZOTAL	The most lateral point of the medial side of the Main olfactory bulb, inner plexiform layer
OLF2	[57, 138, 44]	Accessory olfactory bulb, granular layer	196	HORIZONTAL	The most rostral/proximal point of the Accessory olfactory bulb, granular layer
OLF3	[94, 238, 33]	Main olfactory bulb, granule layer	220	SAGITTAL	The most ventral point of the Accessory olfactory bulb, granular layer
OLF4	[52, 166, 13]	Main olfactory bulb, glomerular layer	212	HORIZONTAL	The most lateral point of the medial side of the Main olfactory bulb, glomerular layer
OLF5	[22, 136, 15]	Main olfactory bulb, granule layer	220	HORIZONTAL	The most ventral point of the dorsal part of the Main olfactory bulb, granule layer
HIP1	[301, 61, 61]	Field CA1, pyramidal layer	407	CORONAL	The most dorsal point of the Field CA1, pyramidal layer
HIP2	[245, 97, 27]	Field CA3, pyramidal layer	495	HORIZONTAL	The most rostral point of the Field CA3, pyramidal layer
HIP3	[333, 155, 119]	Field CA3, pyramidal layer	495	SAGITTAL	The center of the merging between the two parts of the Field CA3, pyramidal layer
HIP4	[359, 127, 108]	Dentate gyrus, granule cell layer	632	SAGITTAL	The most caudal point of the dorsal Dentate gyrus, granule cell layer after the separation
HIP5	[359, 156, 153]	Field CA1, pyramidal layer	407	SAGITTAL	The most caudal point of the Field CA1, pyramidal layer before the breaking of the circle it forms
STR1	[253, 101, 94]	Caudoputamen	672	SAGITTAL	The most dorsal point of the Caudoputamen
STR2	[204, 235, 99]	Caudoputamen	672	CORONAL	The most ventral point of the Caudoputamen
STR3	[250, 230, 78]	Central amygdalar nucleus, medial part	559	CORONAL	The most medial point of the Central amygdalar nucleus, medial part
STR4	[175, 264, 56]	Nucleus accumbens	56	HORIZONTAL	The most ventral point of the Nucleus accumbens
STR5	[152, 216, 4]	Nucleus accumbens	56	SAGITTAL	The center point of the Nucleus accumbens between the two parts of the anterior commissure, olfactory limb just before the latter joins into one single peace
CB1	[484, 195, 118]	Paramedian lobule, granular layer	10681	SAGITTAL	The most ventral point of the Paramedian lobule, granular layer
CB2	[456, 49, 67]	Simple lobule, granular layer	10672	SAGITTAL	The most dorsal point of the Simple lobule, granular layer
CB3	[501, 90, 93]	Crus 2, granular layer	10678	SAGITTAL	The most dorsal point of the Crus 2, granular layer
CB4	[448, 259, 149]	Paraflocculus, granular layer	10687	SAGITTAL	The most ventral point of the Paraflocculus, granular layer
BS1	[412, 195, 61]	Motor nucleus of trigeminal	621	SAGITTAL	The most dorsal point of the Motor nucleus of trigeminal
BS2	[334, 165, 54]	Anterior pretectal nucleus	215	SAGITTAL	The most ventral point of the Anterior pretectal nucleus
BS3	[429, 204, 13]	Abducens nucleus	653	SAGITTAL	The most dorsal point of the Abducens nucleus
BS4	[435, 271, 74]	Facial motor nucleus	661	HORIZONTAL	The most lateral point of the Facial motor nucleus
BS5	[395, 236, 13]	Tegmental reticular nucleus	574	SAGITTAL	The most caudal point of the Tegmental reticular nucleus
CTX1	[239, 237, 117]	Basolateral amygdalar nucleus, anterior part	303	HORIZONTAL	The most rostral point of the Basolateral amygdalar nucleus, anterior part
CTX2	[384, 88, 115]	Primary visual area, layer 6b	305	HORIZONTAL	The most caudal point of the Primary visual area, layer 6b
TH1	[274, 111, 62]	Lateral dorsal nucleus of thalamus	155	SAGITTAL	The most dorsal point in the Lateral dorsal nucleus of thalamus
TH2	[269, 131, 84]	Reticular nucleus of the thalamus	262	SAGITTAL	The most dorsal point in the Reticular nucleus of the thalamus
TH3	[258, 153, 41]	Anteroventral nucleus of thalamus	255	SAGITTAL	The most caudal point of the Anteroventral nucleus of thalamus

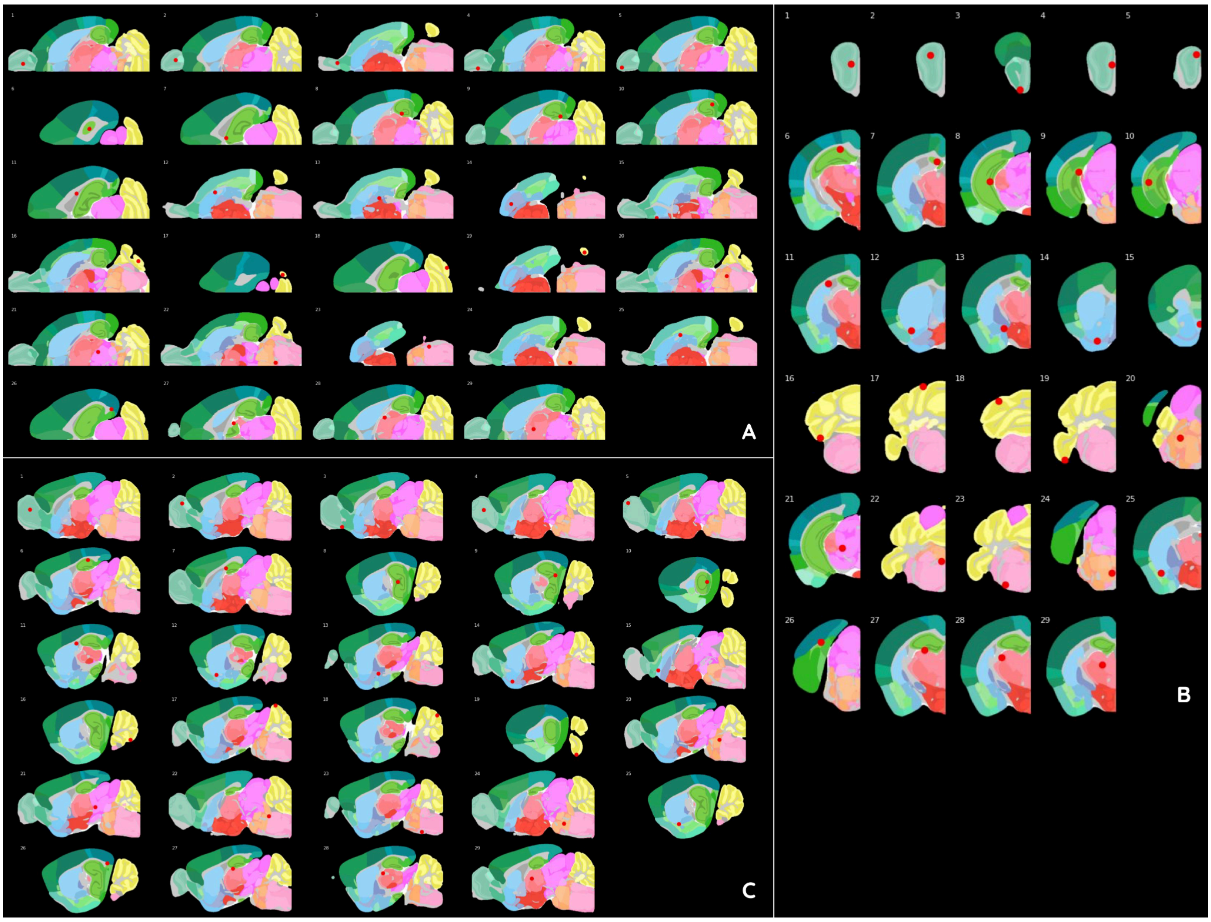

FIGURE S7.2 | Plot of the 29 fiducial anatomical points of interest in the ANNOTv3 in the three conventional incidences (A) horizontal, (B) coronal, and (C) sagittal.

S7.2 Detailed Results

The detailed results of the Target Registration Error (TRE) calculated before and after the alignment are presented in **Figure S7.3**. Among the 29 fiducial points, three were considered to have insufficient confidence (STR2, STR3 and TH2; **Figure S7.3B**) and one was identified as an overlay (BS2 for operator 3: **Figure S7.3C**). Those three points (STR2, STR3, and BS2) were removed from the final average estimation, resulting in a 26 points dataset.

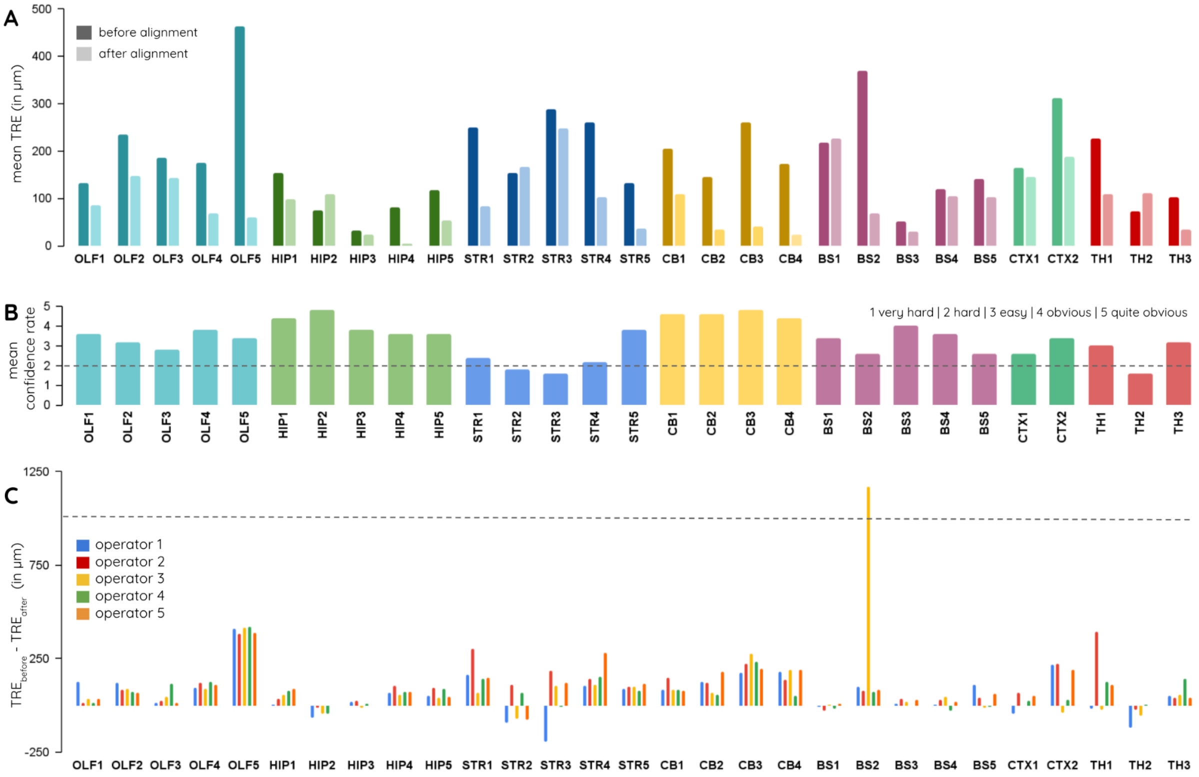

FIGURE S7.3 | Detailed results for the point identification in the ARA Nissl$_{COR}$ before (ARA Nissl$_{COR}$) and after (ARA$_{BBP}$ Nissl$_{COR}$; after Step A5) alignment in the CCFv3. (A) Mean TRE calculated between the target (operator) and the reference (fiducial) point before (dark) and after (light) alignment, (B) mean confidence rate given by operators, and (C) TRE difference before and after the alignment for each of the 5 operators.

S8 Detailed qualitative analysis of the alignment of the ARA Nissl$_{COR}$ in the CCFv3

S8.1 Aligning the ARA Nissl$_{COR}$ in the CCFv3

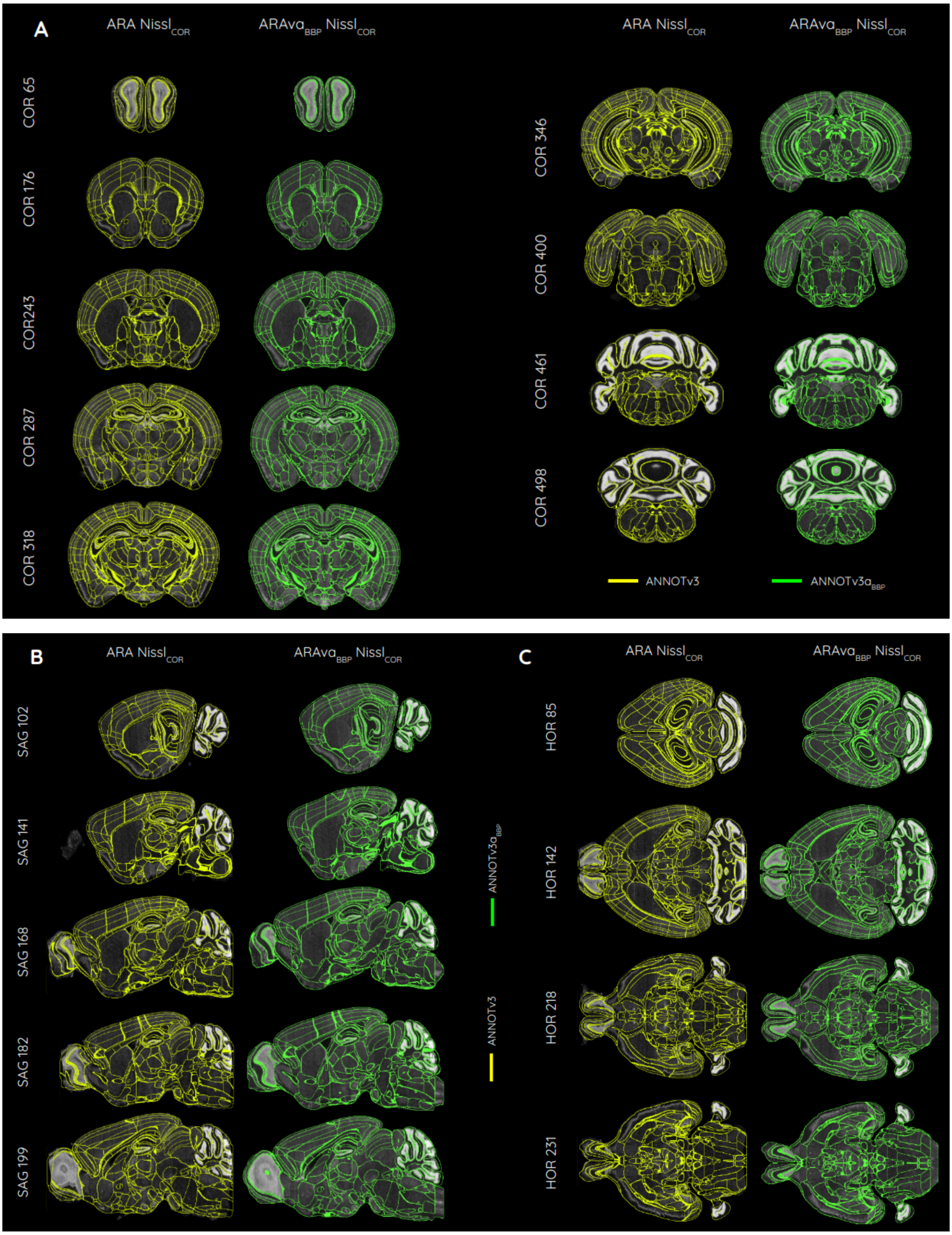

FIGURE S8.1 | Overview of the superimposition of the annotations on the Nissl anatomy for the ARA Nissl$_{COR}$ (raw) with ANNOTv3 (yellow), and ARAva$_{BBP}$ Nissl$_{COR}$ (our alignment) with ANNOTv3a (green) in the (A) coronal, (B) sagittal, and (C) horizontal incidence.

S8.2 Aligning the AIBS $Nissl_{SAG}$ and WAXH $Nissl_{HOR}$ with the $ARAva_{BBP}$ $Nissl_{COR}$

The AIBS $Nissl_{SAG}$ and the WAXH $Nissl_{HOR}$ were both aligned to the $ARAva_{BBP}$ $Nissl_{COR}$ using the NiftyRefF3D registration algorithm, resulting into the $AIBS_{BBP}$ $Nissl_{SAG}$ and the $WAXH_{BBP}$ $Nissl_{HOR}$ (**Figure S8.2**).

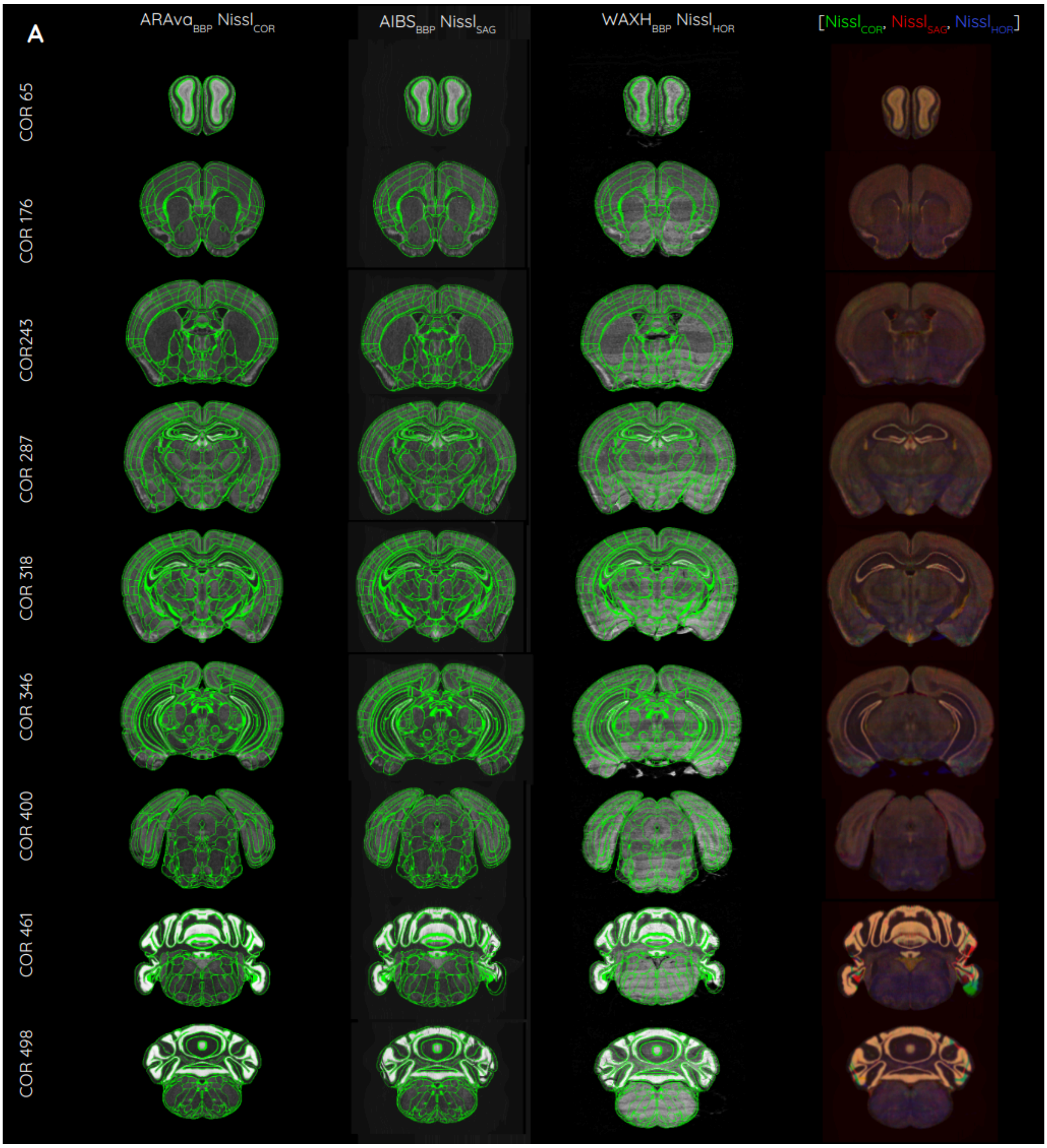

FIGURE S8.2 | Overview of the three aligned volumes in the CCFv3 at 25 µm isotropic resolution: $ARAva_{BBP}$ $Nissl_{COR}$, $AIBS_{BBP}$ $Nissl_{SAG}$, and $WAXH_{BBP}$ $Nissl_{HOR}$ with the $ANNOTv3a_{BBP}$ boundaries overlayed (in green), plus, in the last column, the $ARAva_{BBP}$ $Nissl_{COR}$ is overlaid (in green), $AIBS_{BBP}$ $Nissl_{SAG}$ (in red), and $WAXH_{BBP}$ $Nissl_{HOR}$ (in blue) in the (A) coronal, (B) sagittal, and (C) horizontal incidence.

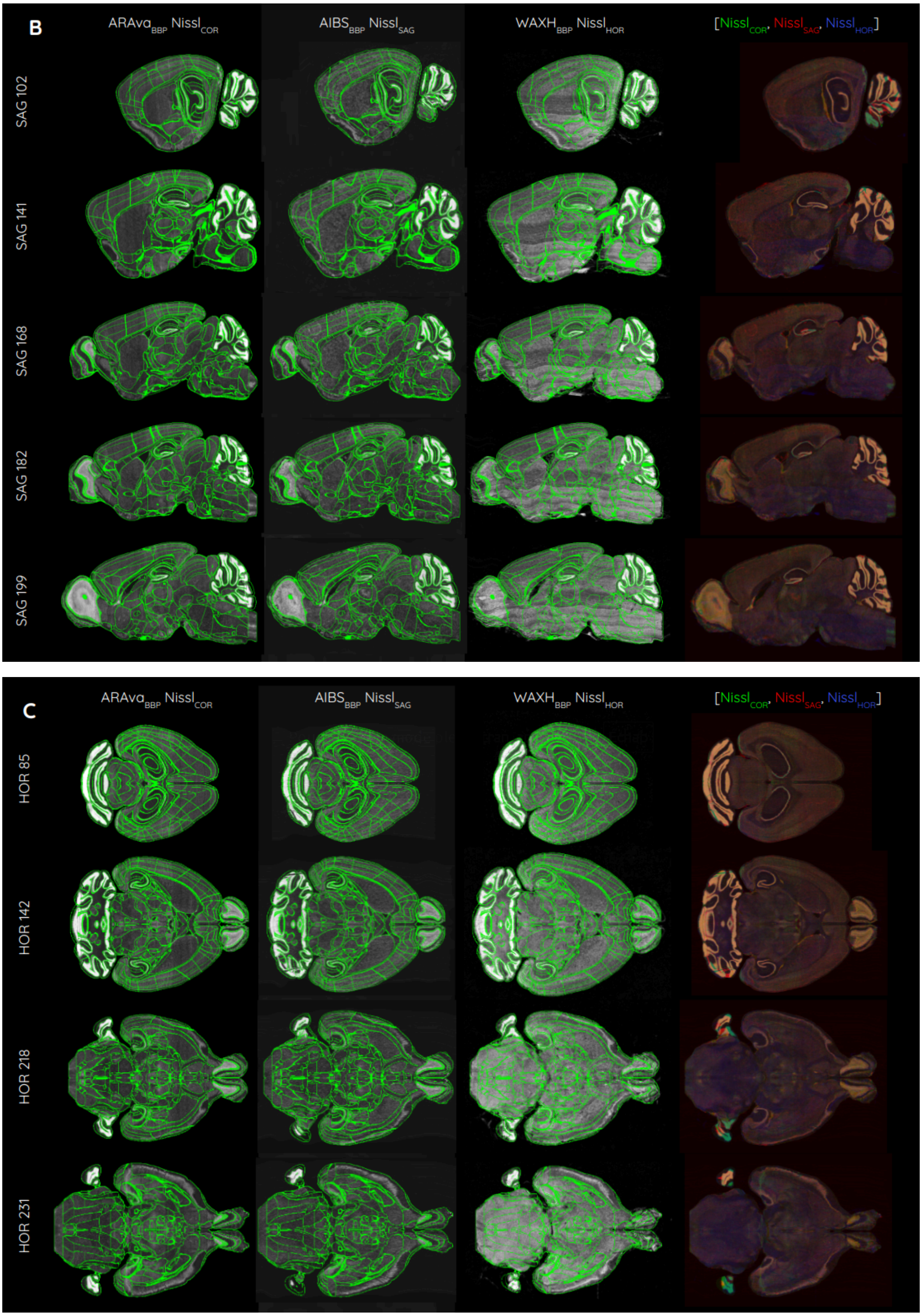
B
ARAva BBP Nissl COR
AIBS BBP Nissl SAG
WAXH BBP Nissl HOR
[Nissl COR, Nissl SAG, Nissl HOR]
SAG 102
SAG 141
SAG 168
SAG 182
SAG 199
C
ARAva BBP Nissl COR
AIBS BBP Nissl SAG
WAXH BBP Nissl HOR
[Nissl COR, Nissl SAG, Nissl HOR]
HOR 85
HOR 142
HOR 218
HOR 231

S9 Labels added in the cerebellum and artifact correction

We provide here an update on the cerebellum hierarchy in **Figure S8.1**, including 16 lobules: lingula (I), lobule (II), lobule (III), lobules (IV-V), declive (VI), folium-tuber vermis (VII), pyramus (VIII), uvula (IX), nodulus (X), simple lobule, crus 1, crus 2, paramedian lobule, copula pyramidis, paraflocculus, and flocculus. These 16 lobules each have 3 children defined in the hierarchy: granular layer, molecular layer and Purkinje layer. The ANNOTv3 provided by the Allen Institute does not include these regions. In the new version of the atlas we proposed, we produced the annotation for the two first ones (granular and molecular layers) at 25 µm isotropic resolution, plus the last one (Purkinje layer) at the junction between the granular and molecular layer of each lobule in the 10 µm isotropic resolution.

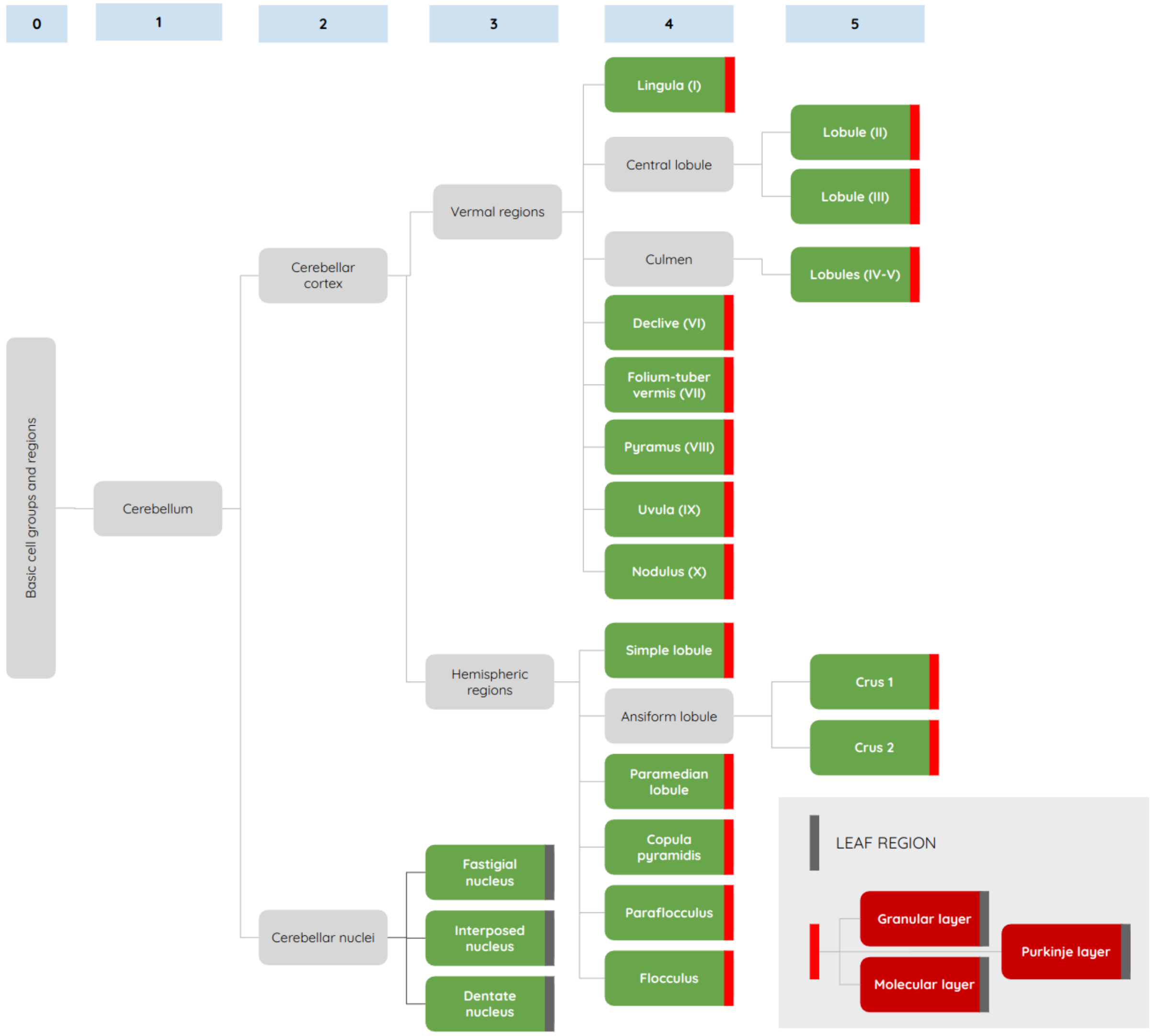

FIGURE S9.1 | Cerebellum hierarchy, focusing on regions which are missing in the CCFv3 annotation (in red).

Based on established cell density patterns in the cerebellar layers (Šišková et al., 2013; Hayashi et al., 2017), we estimated the average cell density within the granular and molecular layers independently and applied automated Otsu thresholding (Otsu, 1975) to

detect and correct outlier voxels indicative of artifacts. The procedure aimed to enhance the lowest intensity values within the granular layer and reduce the highest intensity values within the molecular layer, as these abnormalities corresponded to tearing or staining artifacts. This approach relied on the assumption that the anatomical annotation accurately corresponded to the Nissl-stained tissue in the cerebellum.

Low-intensity voxels in the granular layer were increased toward the mean intensity of the other non-zero voxels in the layer, followed by a smoothing step to ensure continuity. Conversely, high-intensity voxels in the molecular layer were reduced toward the mean intensity of the other non-zero voxels in the layer, with smoothing applied to the adjusted values. This automated correction process improved the visual and quantitative consistency of the cerebellar layers in the Nissl volume (**Figure S9.2A**).

The results presented in **Figure S9.2B** clearly demonstrate a more realistic and homogeneous appearance within the cerebellar layers, with the granular layer (characterized by high cell density) and the molecular layer (with lower cell density) exhibiting their expected structural properties. The prominent tear artifacts observed in the ARA $Nissl_{COR}$ and $ARAva_{BBP}$ $Nissl_{COR}$ volumes were substantially reduced, resulting in a more accurate and anatomically consistent representation of the cerebellum.

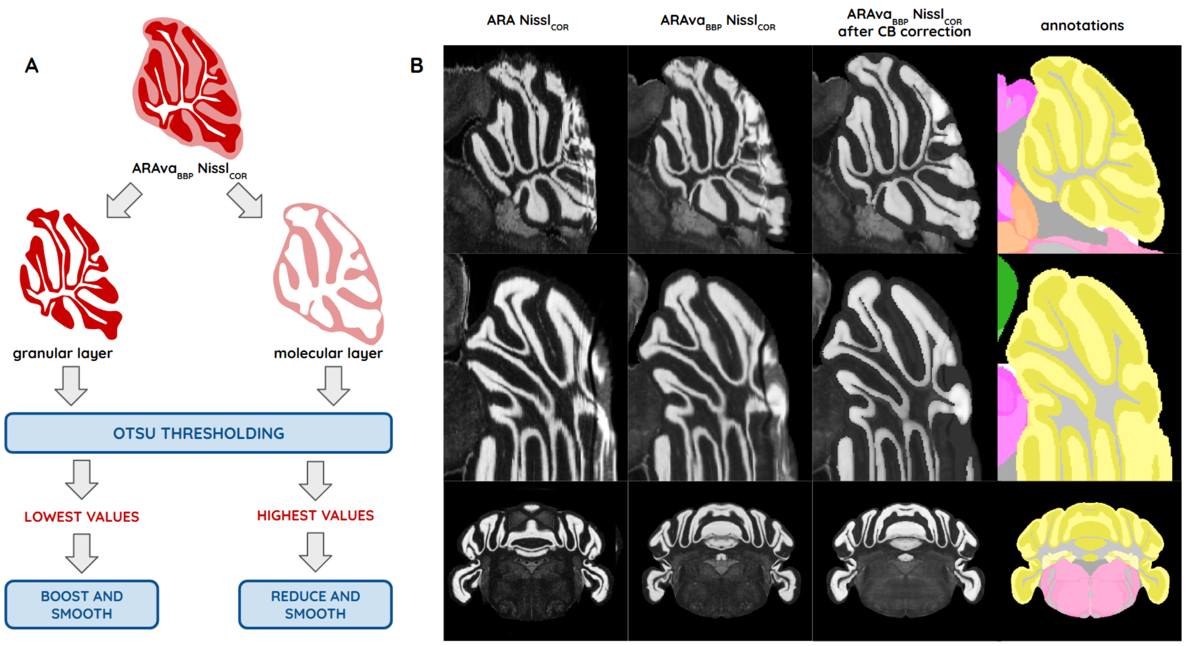

FIGURE S9.2 | Correction of the tear artifacts in the cerebellum, with (A) the automated method implemented for the correction, and (B) the results of the corrected $ARAva_{BBP}$ $Nissl_{COR}$ compared to the ARA $Nissl_{COR}$ and non corrected $ARAva_{BBP}$ $Nissl_{COR}$ versions.
